# Diagnosing and Predicting Mixed-Culture Fermentations with Unicellular and Guild-Based Metabolic Models

**DOI:** 10.1128/mSystems.00755-20

**Published:** 2020-09-29

**Authors:** Matthew J. Scarborough, Joshua J. Hamilton, Elizabeth A. Erb, Timothy J. Donohue, Daniel R. Noguera

**Affiliations:** a The Great Lakes Bioenergy Research Center, UW-Madison, Madison, Wisconsin, USA; b Department of Civil and Environmental Engineering, UW-Madison, Madison, Wisconsin, USA; c Department of Civil and Environmental Engineering, University of Vermont, Burlington, Vermont, USA; d Department of Biochemistry, UW-Madison, Madison, Wisconsin, USA; e Department of Chemical and Biological Engineering, UW-Madison, Madison, Wisconsin, USA; f Department of Bacteriology, UW-Madison, Madison, Wisconsin, USA; Institute for Genomics & Systems Biology

**Keywords:** carboxylate platform, community modeling, medium-chain carboxylic acids, medium-chain fatty acids, microbiome, mixed-culture fermentation, octanoic acid

## Abstract

Multispecies microbial communities determine the fate of materials in the environment and can be harnessed to produce beneficial products from renewable resources. In a recent example, fermentations by microbial communities have produced medium-chain fatty acids (MCFAs). Tools to predict, assess, and improve the performance of these communities, however, are limited. To provide such tools, we constructed two metabolic models of MCFA-producing microbial communities based on available genomic, transcriptomic, and metabolomic data. The first model is a unicellular model (iFermCell215), while the second model (iFermGuilds789) separates fermentation activities into functional guilds. Ethanol and lactate are fermentation products known to serve as substrates for MCFA production, while acetate is another common cometabolite during MCFA production. Simulations with iFermCell215 predict that low molar ratios of acetate to ethanol favor MCFA production, whereas the products of lactate and acetate coutilization are less dependent on the acetate-to-lactate ratio. In simulations of an MCFA-producing community fed a complex organic mixture derived from lignocellulose, iFermGuilds789 predicted that lactate was an extracellular cometabolite that served as a substrate for butyrate (C4) production. Extracellular hexanoic (C6) and octanoic (C8) acids were predicted by iFermGuilds789 to be from community members that directly metabolize sugars. Modeling results provide several hypotheses that can improve our understanding of microbial roles in an MCFA-producing microbiome and inform strategies to increase MCFA production. Further, these models represent novel tools for exploring the role of mixed microbial communities in carbon recycling in the environment, as well as in beneficial reuse of organic residues.

**IMPORTANCE** Microbiomes are vital to human health, agriculture, and protecting the environment. Predicting behavior of self-assembled or synthetic microbiomes, however, remains a challenge. In this work, we used unicellular and guild-based metabolic models to investigate production of medium-chain fatty acids by a mixed microbial community that is fed multiple organic substrates. Modeling results provided insights into metabolic pathways of three medium-chain fatty acid-producing guilds and identified potential strategies to increase production of medium-chain fatty acids. This work demonstrates the role of metabolic models in augmenting multi-omic studies to gain greater insights into microbiome behavior.

## Abstract (precis)

Microbiomes are vital to human health, agriculture, and protecting the environment. Predicting behavior of self-assembled or synthetic microbiomes, however, remains a challenge. In this work, we used unicellular and guild-based metabolic models to investigate production of medium-chain fatty acids by a mixed microbial community that is fed multiple organic substrates. Modeling results provided insights into metabolic pathways of three medium-chain fatty acid-producing guilds and identified potential strategies to increase production of medium-chain fatty acids. This work demonstrates the role of metabolic models in augmenting multi-omic studies to gain greater insights into microbiome behavior.

## INTRODUCTION

Mixed microbial fermentations have benefited humanity throughout history (1). Since the mid-18th century, mixed-culture fermentations have produced valuable chemicals and recovered energy from wastes (2). Many mixed microbial fermentations involve the synergistic activities of several functional groups to convert organic substrates into valuable products. In anaerobic digestion, fermentation products like acetic acid (C2) and H_2_ are used to support microbial production of methane, thus enabling the recovery of a large fraction of the chemical energy originally present in the complex organic substrates. While anaerobic digestion technologies are well established, other strategies to expand the range of potential products of mixed-culture fermentations are only beginning to emerge. One example of such a mixed-culture fermentation is the carboxylate platform, which produces medium-chain fatty acids (MCFAs) as a potentially valuable set of bio-based products (3).

MCFAs are attractive carboxylate platform products because of their many industrial uses (4). Octanoic acid (C8), an 8-carbon linear monocarboxylic acid, is a particularly valuable MCFA because of its high market value and relative ease of recovery. Further, when C8 is reduced to its corresponding alkane, it can substitute for octane in liquid transportation fuels. Recent research has demonstrated the feasibility of using mixed-culture fermentations to transform renewable resources into MCFAs (5–7). While C8 is produced by some self-assembled bioreactor microbial communities, hexanoic acid (C6) is often the primary MCFA, along with two short-chain fatty acids (SCFAs), acetic (C2) and butyric (C4) acids (5–10).

The microbial metabolic networks involved in converting complex organic substrates to MCFAs by mixed-culture fermentations are not fully elucidated. Consequently, there is a lack of knowledge on how to obtain a carboxylate platform community that produces C8 as the main MCFA, or how to minimize the accumulation of SCFAs. Initial models of mixed-culture fermentations to produce products other than methane have focused on simulating metabolic networks for production of C2, C4, propionic acid, ethanol, lactic acid, and H_2_ from glucose (11). Subsequent models took into account that multiple electron carriers (e.g., ferredoxin, NADH) play different roles in these metabolic networks, enabling the simulation of energy-conserving electron-bifurcating reactions during production of C4 by reverse β-oxidation (12). While a recent model considered the role of homoacetogens as an H_2_-consuming member of mixed-culture fermentation, it was limited to producing C4 as the major product of reverse β-oxidation and did not include MCFA production (13). Additionally, recent metabolic models assessed the potential for producing C2, C3, C4, and valerate (C5) from amino acids (14). While all of these models have enabled predictions of mixed-culture fermentations from a single organic substrate, they lack the ability to simulate production of MCFAs from complex feedstocks.

**FIG 1:**
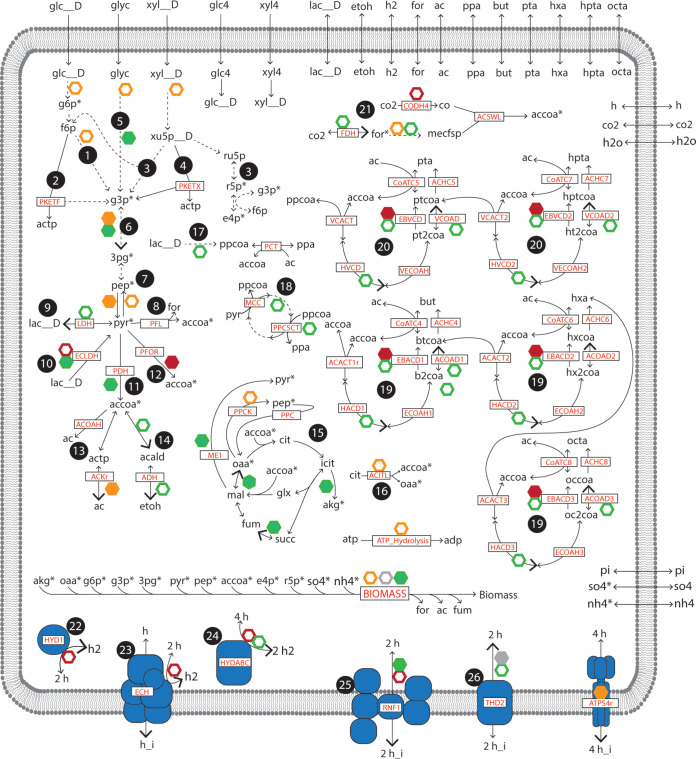
Summary of metabolic networks in iFermCell215. Metabolites with an asterisk are required for biomass production. Open hexagons indicate consumption of ATP (orange), NADH (green), NADPH (gray), and reduced ferredoxin (red). Closed hexagons indicate production of these compounds and consumption of ADP (orange), NAD^+^ (green), NADP^+^ (gray), and oxidized ferredoxin (red). For reversible reactions, larger arrowheads indicate the reaction directionality corresponding with consumption and/or production of ATP, NADH, NADPH, and reduced ferredoxin. Dashed lines indicate multiple reactions. Key reactions are indicated according to the abbreviations used in iFermCell215 (Data Set S3). Pathways are identified by number: (1) upper glycolysis; (2) phosphoketolase metabolism of fructose; (3) nonoxidative pentose phosphate pathway; (4) phosphoketolase metabolism of xylulose; (5) glycerol utilization; (6) lower glycolysis; (7) gluconeogenesis; (8) pyruvate formate lyase; (9) lactate metabolism; (10) pyruvate flavodoxin oxidoreductase; (11) pyruvate dehydrogenase; (12) acetate metabolism; (13) ethanol metabolism; (14) incomplete citric acid cycle with glyoxylate shunt; (15) anaplerotic metabolism of phosphoenolpyruvate, pyruvate, and oxaloacetate; (16) ATP citrate lyase; (17) propionate production via the acryloyl-CoA pathway; (18) propionate production via the methylmalonyl-CoA pathway; (19) even-chain reverse β-oxidation; (20) odd-chain reverse β-oxidation; (21) homoacetogenesis via the Wood-Ljungdahl pathway; (22) ferredoxin-dependent hydrogenase; (23) proton-translocating hydrogenase; (24) electron-confurcating hydrogenase; (25) proton-translocating ferredoxin:NAD^+^ oxidoreductase; (26) NAD(P) transhydrogenase. For clarity, not all reactions or metabolites are shown. More information on the reactions and metabolites contained in iFermCell215 is provided in Data Set S4.

**FIG 2:**
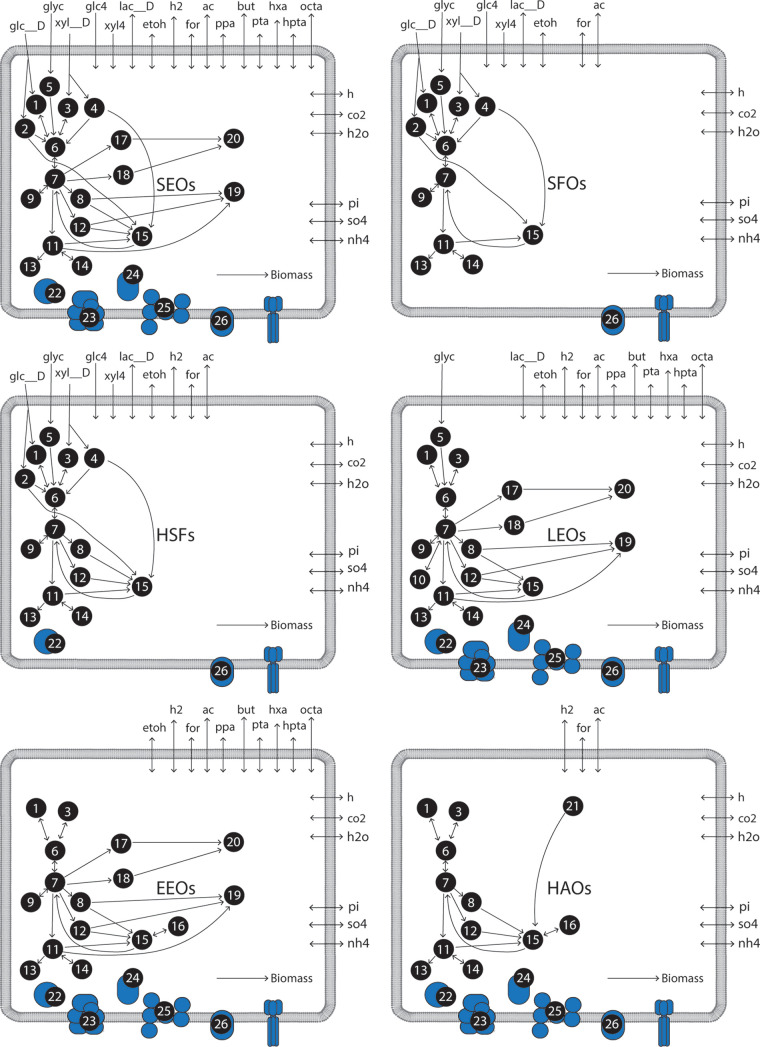
Summary of metabolic networks for six functional guilds contained in iFermGuilds789. Refer to Fig. 1 for details on pathways included in the iFermGuilds789 model. More information on the reactions and metabolites contained in the iFermGuilds789 model is provided in Data Set S4.

In this study, we constructed a unicellular model (iFermCell215) and a guild-based microbial community model (iFermGuilds789) that offer expanded substrates and metabolic pathways compared to existing models. We used iFermCell215 and iFermGuilds789 to analyze pathways that lead to MCFA production. Simulations were performed with single organic substrates, including sugars, glycerol, lactate, and acetate, with ethanol and acetate or lactate and acetate as cosubstrates, and with complex organic mixtures. The iFermGuilds789 model was also used to evaluate predictions of observed bioreactor behavior and of the flow of carbon and electrons through different guilds within the mixed microbial community. In total, results of individual model simulations generated new hypotheses on how to increase MCFA production by mixed microbial communities. The metabolic networks in iFermCell215 and those in iFermGuilds789 are shown in Fig. 1 and 2, respectively.

## RESULTS AND DISCUSSION

### Simulation of mixed-culture fermentations with iFermCell215.

We first investigated predictions derived from an iFermCell215 model under conditions where single substrates were consumed within this unit. The iFermCell215 model depicts a simplified representation of fermentation in a mixed microbial community where interspecies transfer of metabolites is assumed to have no energetic penalty and is similar to previously published models of mixed-culture fermentations (11–13). For this analysis, we required that all possible solutions released ≥50 kJ per mol ATP produced (15).

**FIG 3:**
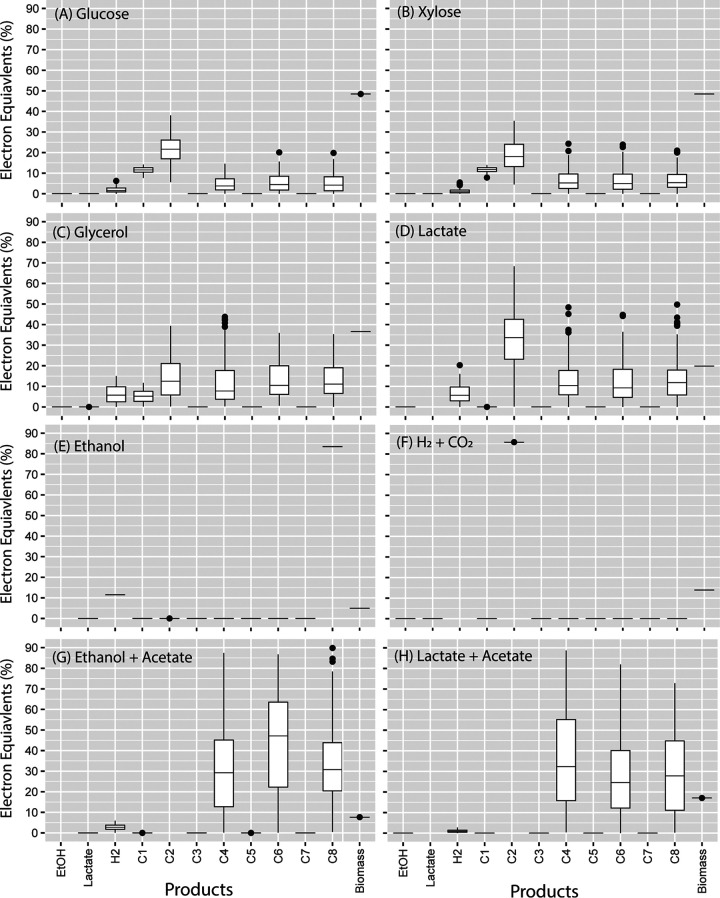
Flux sampling results for iFermCell215 with objective function to optimize biomass production when fed substrates indicated within the plots: glucose (A), xylose (B), glycerol (C), lactate (D), ethanol (E), and H_2_ and CO_2_ (with H_2_ as the limiting substrate) (F). Box plots show the results of 101 simulations. Median values are indicated by a horizontal line within the box, and the top and bottom of the box indicate the 25th and 75th percentiles, respectively. Whiskers indicate values within 1.5 times the interquartile range, and closed circles indicate values outside this range.

Using flux sampling, iFermCell215 provided 101 solutions that maximized biomass production depending on the substrates provided to the simulation (Fig. 3). For instance, when we modeled the utilization of glucose or xylose as the sole substrate, iFermCell215 predicted multiple solutions that allowed for maximum biomass production (40% of electron equivalents [eeq] are incorporated into biomass) with H_2_, C2, C4, C6, and C8 as predicted products (Fig. 3A). None of the solutions in this flux sampling analysis predicted ethanol or lactate as significant products. The predicted biomass yield (eeq incorporated into biomass per eeq consumed) decreased with glycerol (37%), lactate (20%), and ethanol (5.9%) as sole substrates. Consumption of ethanol as a sole substrate was predicted to produce C8 and H_2_ as the most energetically favorable solution (Fig. 3D), whereas consumption of lactate as a sole substrate produced optimal solutions through heterofermentation (Fig. 3C) and consumption of H_2_ and CO_2_ was predicted to produce C2 for optimal biomass growth, which is consistent with the activity of homoacetogens (Fig. 3E). These results suggest that sugars, glycerol, and lactate can produce significant amounts of multiple fermentation products, including C2, C4, C6, and C8, while supporting optimal biomass production and that production of ethanol or lactate, the two common fermentation products described as intermediates in MCFA production (16), are not as energetically favorable as producing other fermentation products. It further suggests that consumption of ethanol as a sole substrate should favor production of C8 and, therefore, that ethanol may be a better intermediate product for optimizing C8 production than lactate. However, since the fermentation of complex organic substrates by mixed communities often results in accumulation of multiple fermentation products, we also simulated conditions under which ethanol and acetate or lactate and acetate were cosubstrates for MCFA production.

### Simulation of ethanol and acetate utilization with iFermCell215.

Ethanol is a substrate that has often been used for chain elongation (17), and early work on MCFA production was performed using the ethanol- and acetate-consuming Clostridium kluyveri (18, 19). During initial work with *C. kluyveri*, it was proposed that C4 could be produced from two moles of ethanol, without using acetate as an electron acceptor (19). Recent work has confirmed that *C. kluyveri* produces MCFAs from ethanol as a sole substrate in batch experiments (20), in agreement with the iFermCell215 prediction (Fig. 3E). Production of MCFAs from C2 as a sole substrate has not been observed and is not predicted by iFermCell215. Past studies of ethanol elongation have investigated optimal ratios of ethanol-to-acetate for MCFA production (17, 21, 22). Here, we use iFermCell215 to assess impacts of different molar ratios of acetate to ethanol (C2:EtOH) on predicted product formation.

**FIG 4:**
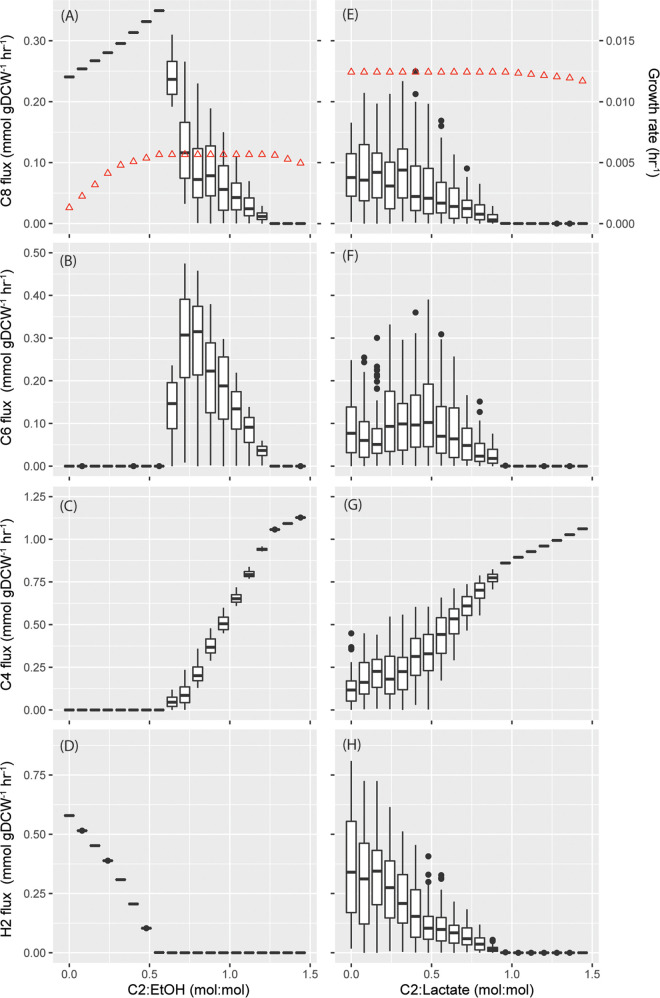
Flux sampling results for iFermCell215 when consuming ethanol and acetate (A to D) or lactate and acetate (E to H) at different molar ratios of acetate to ethanol (C2:EtOH) or acetate to lactate (C2:Lactate). Results are shown for fluxes of octanoate (C8), hexanoate (C6), butyrate (C4), and H_2_. These results are shown as box plots, as described previously (Fig. 3), representing the results of 101 simulations for each modeled ratio. The predicted growth rate (h^−1^) is shown for ethanol (A) and lactate (E) as red triangles plotted on the secondary *y* axis.

When supplied with both ethanol and C2, the iFermCell215 predicted growth rate of 0.11 h^−1^ (Fig. 4A) is more than double the predicted growth rate with ethanol as the sole substrate (Fig. 4A, C2:EtOH = 0), suggesting an ecological advantage for microorganisms that can coutilize ethanol and acetate. This growth rate can be achieved at C2:EtOH ratios from 0.56 to 1.28 mol:mol. At ratios of 0.56 mol:mol and lower, C8 and H_2_ are the only major predicted products (Fig. 4, left panels). At ratios between 0.56 and 1.20, a mixture of C8, C6, and C4 is predicted by iFermCell215, and at ratios of 1.28 and above, C4 is predicted as the sole product. These model predictions are consistent with previous experimental work that shows that low ratios of C2:EtOH improve specificity of C8 production (21). The overall production rate of MCFAs, however, has been shown to improve at higher ratios of C2:EtOH (21). The highest overall MCFA production rate was achieved by Spirito et al. at a ratio of 0.53 mol:mol (21), which is very close to the model-predicted ratio of 0.56 that optimizes biomass growth and C8 production. The production of H_2_ at low ratios of C2:EtOH raises the question as to the impact of H_2_ partial pressure on the thermodynamic feasibility of the model results. We used iFermCell215 to calculate the maximum H_2_ partial pressure for all 101 scenarios and found that the lowest allowable H_2_ partial pressure for growth is greater than 10^6^ atm (see Fig. S1 in the supplemental material), indicating that the proposed transformations are thermodynamically favorable under all bioreactor H_2_ partial pressure conditions.

10.1128/mSystems.00755-20.2 FIG S1 Maximum partial pressures for the iFermCell215 results predicted with acetate and ethanol as cosubstrates (A) and acetate and lactate as cosubstrates (B). Values greater than 10^9^ atm are not shown. These values correspond to the scenarios plotted in Fig. 4. Download FIG S1, PDF file, 0.1 MB. Copyright © 2020 Scarborough et al. 2020 Scarborough et al. This content is distributed under the terms of the Creative Commons Attribution 4.0 International license.

In total, iFermCell215 predicts that the C2:EtOH ratio either could be used to improve production of C8 as a desired product or could be an indication that the environmental conditions in a bioreactor would lead to a mixture of fermentation products. At low C2:EtOH ratios (<0.56), iFermCell215 predicts that C8 should be the abundant product. At medium C2:EtOH ratios (0.56 to 1.20), iFermCell215 predicts that a mixture of C4, C6, and C8 would be produced, while at high ratios (>1.20) of these substrates, iFermCell215 predicts C4 would be the predominant product. Therefore, in a microbial community fermenting a mixture of substrates, iFermCell215 predicts that different ratios of ethanol and C2 will impact the composition and abundance of products. Additionally, iFermCell215 predicts that, if feeding a reactor with ethanol and C2, attention should be paid to creating a feeding regime that optimizes production of the target product.

### Simulation of lactate and acetate utilization with iFermCell215.

In addition to ethanol, lactate is often used as a substrate for microbial chain elongation. The well-studied Megasphaera elsdenii has been shown to produce MCFAs from lactate (23), and recent chain elongation studies with microbial communities have explored lactate as both a substrate (9, 24) and a proposed key intermediate that supports chain elongation from carbohydrate fermentation (25, 26). While ethanol and lactate are both potential fermentation products and substrates for chain elongation, iFermCell215 predicts that the pathways for utilization of these substrates have key differences that impact the terminal fermentation products, as shown in the flux sampling analyses (Fig. 3D and E), and may impact the products when C2 is a cosubstrate. Therefore, similar to the analysis shown above for C2:EtOH ratios, we used iFermCell215 to assess impacts of different C2-to-lactate ratios.

In the case of lactate, the predicted growth rate does not change when C2 is a cosubstrate (Fig. 4E). This suggests that, unlike ethanol and C2 coutilization, there is no apparent energetic benefit (reflected in growth rate) for a microorganism to coutilize lactate and C2. Low ratios of C2:lactate are predicted by iFermCell215 to produce a variety of end products, including C8, C6, C4, and H_2_ (Fig. 4, right panels). As the C2:lactate ratio increases, the product distribution is predicted by iFermCell215 to change, with a decrease in MCFA and H_2_ production accompanied by an increase in C4 production, and at ratios of 0.96 and above, C4 is predicted as the sole product. Like ethanol and C2 coutilization, lactate and C2 coutilization is not predicted by iFermCell215 to be impacted by bioreactor H_2_ partial pressure (Fig. S1). These results suggest that when lactate is a key intermediate in an MCFA-producing community, controlling the relative amounts of C2 and lactate may not be an effective strategy for increasing C6 and C8 production. However, as with ethanol, an excess amount of acetate is predicted by iFermCell215 to increase C4 production.

The predicted differences in product formation when acetate is cometabolized with ethanol or lactate suggest important differences in the metabolism of these substrates. Therefore, to understand these differences, we also used iFermCell215 to assess the predicted metabolic pathways of ethanol and lactate utilization.

### Predicted metabolic pathways for ethanol and lactate utilization.

**FIG 5:**
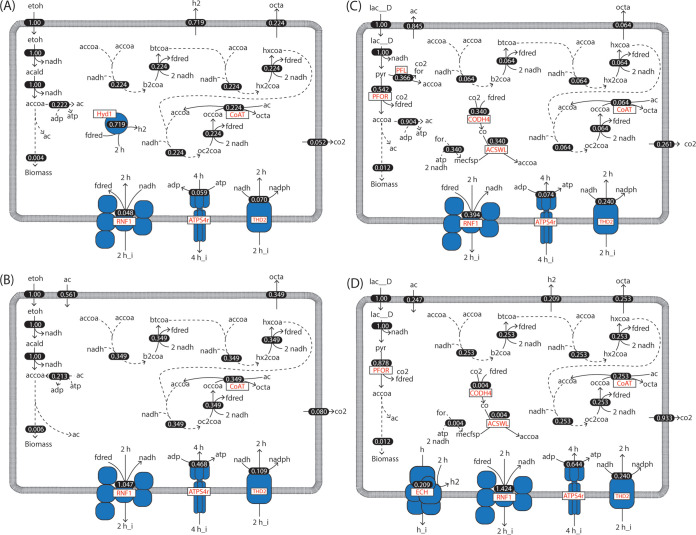
Predicted pathways of ethanol utilization by iFermCell215 using pFBA when ethanol is the sole substrate (A), ethanol and acetate are cosubstrates (B), lactate is a sole substrate (C), and lactate and acetate are cosubstrates (D). Flux values (shown as white text) have units of mmol gDCW^−1^ h^−1^. For both scenarios, the model was constrained to take up 1 mmol gDCW^−1^ h^−1^ of ethanol or 1 mmol gDCW^−1^ h^−1^ of lactate. For scenarios with acetate coutilization, acetate was provided in excess. Key reactions indicated by red text include a ferredoxin hydrogenase (Hyd1), a CoA transferase (CoAT), a proton-translocating ferredoxin:NAD^+^ oxidoreductase (RNF1), ATP synthase (ATPS4r), NADH:NADP transhydrogenase (THD2), and energy-conserving hydrogenase (ECH). Metabolite abbreviations are defined in Data Set S2.

The flux sampling analysis obtained with iFermCell215 consuming ethanol as a sole substrate converged to a solution in which C8 was the only significant fermentation product (Fig. 3E). The flux of electron equivalents in this simulation predicted that reactions catalyzed by ferredoxin hydrogenase (Hyd1), proton-translocating ferredoxin:NAD^+^ oxidoreductase (also known as *Rhodobacter* nitrogen fixation complex [RNF]), a coenzyme A transferase (CoAT), and acetate kinase (AckR) were key enzymes used in the network (Fig. 5A). The genome of the well-studied ethanol chain-elongating organism, *C. kluyveri*, is predicted to contain genes that encode the proteins for catalyzing all of these reactions (27). Further, Hyd1 is the only hydrogen-producing gene annotated in the genome of *C. kluyveri*.

The flux through the above-described enzymes predicts extensive use of energy-conserving mechanisms that rely on the generation of an ion-motive force (IMF). When ethanol is the sole substrate (Fig. 5A), iFermCell215 predicted that the IMF is generated by the ATP synthase while hydrolyzing ATP. The model predicts that the RNF enzyme uses the IMF to generate reduced ferredoxin and that ATP is provided by acetate kinase. The reverse β-oxidation cycle uses an electron-bifurcating acyl coenzyme A (acyl-CoA) dehydrogenase that also generates reduced ferredoxin, and redox balance is maintained by Hyd1 that uses the reduced ferredoxin to generate H_2_ (Fig. 5A). When C2 is available in addition to ethanol, iFermCell215 predicts that no net H_2_ production is required for redox balance (Fig. 5B). Instead, iFermCell215 predicts that the activities of several enzymes are reversed, with the RNF complex generating IMF, ATP synthase producing ATP, and acetate kinase consuming ATP to generate acetyl-CoA. Thus, the same set of reactions is predicted to be important by iFermCell215 when using ethanol as a sole substrate or ethanol and C2 as cosubstrates, but the predicted direction of key reactions changes (Fig. 5A and B). These predictions suggest that organisms that elongate ethanol may readily transition between utilizing ethanol as a sole substrate and coutilizing ethanol and C2 since the same enzymes are predicted to be used, and the coutilization of C2 and ethanol improves growth rates (Fig. 4A).

Unlike ethanol utilization, lactate conversion to acetyl-CoA is predicted by iFermCell215 to produce CO_2_, formate, and reduced ferredoxin (Fig. 5C and D). The Wood-Ljungdahl pathway is also predicted by iFermCell215 to use this CO_2_, formate, and reduced ferredoxin to produce C2 (Fig. 5C and D). The predicted ability to generate reduced ferredoxin during lactate consumption (compared to only NADH from ethanol consumption) enables iFermCell215 to utilize a much wider range of pathways for generating energy and maintaining redox balance. The reverse β-oxidation cycle also uses an electron-bifurcating acyl-CoA dehydrogenase under these conditions, generating reduced ferredoxin, with RNF also predicted to be active in redox balancing. These predictions of iFermCell215 explain the wide range of products that are possible from utilization of lactate as sole substrate and coutilization of C2 and lactate. Further, this suggests that limiting the available metabolic pathways can impact the predicted products from lactate. Indeed, removing specific reactions in iFermCell215 impacts the predicted product spectrum (Text S1, Fig. S2). This demonstrates that strategies to improve MCFA production may depend on the available set of microbial transformations and may differ between bioreactor microbial communities.

10.1128/mSystems.00755-20.3 FIG S2 Results from additively disabling reactions in iFermCell215 when feeding lactate. (A) Disabling homoacetogenesis; (B) disabling homoacetogenesis and acetate kinase; (C) disabling homoacetogenesis, acetate kinase, and energy-conserving hydrogenases (Ech and HydABC); (D) disabling homoacetogenesis, acetate kinase, energy-conserving hydrogenases, and CoAT; (E) disabling homoacetogenesis, acetate kinase, and energy-conserving hydrogenases (Ech and HydABC); (F) disabling homoacetogenesis, acetate kinase, energy-conserving hydrogenases, CoAT, and an NADH-dependent lactate dehydrogenase (LDH) which requires the model to use an electron-confurcating lactate dehydrogenase reaction. Download FIG S2, PDF file, 0.2 MB. Copyright © 2020 Scarborough et al. 2020 Scarborough et al. This content is distributed under the terms of the Creative Commons Attribution 4.0 International license.

### Simulating community behavior with iFermCell215 and iFermGuilds789.

**FIG 6:**
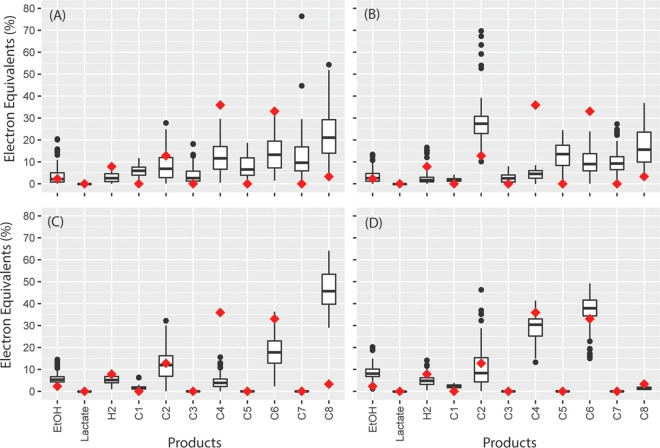
Predicted and observed product formation for a mixed-culture fermentation bioreactor fed lignocellulosic biorefinery residues. (A) Predictions with iFermCell215; (B) predictions with iFermGuilds789; (C) predictions with iFermGuilds789 with reactions knocked out based on metagenomic analyses of the microbial community; (D) predictions with iFermGuilds789 with additional constraints for maximum fluxes through acyl-CoA and CoAT reactions for C6 and C8 production in SEOs and LEOs.

The iFermCell215 model predicted that multiple substrates could lead to C8 production (Fig. 3), but that biomass could be maximized by coproduction of other fermentation products. We wanted to assess if this single-unit model could simulate the observed product yields of a mixed microbial community fed lignocellulosic biorefinery residues, a complex organic substrate (5, 25). The previously described bioreactor was fed lignocellulosic biorefinery residues for 96 days, at which time its microbial community was evaluated using metagenomic and metatranscriptomic analyses along with end product sampling. To represent organic substrate metabolism, the substrate uptake fluxes were set according to the observed consumption of compounds in the feedstock (Table S2). We constrained iFermCell215 by setting the growth rate of the model to 0.0069 h^−1^, corresponding to the bioreactor retention time of 6 days, and we again constrained the model only by requiring a minimum of 50 kJ of free energy release per mol of ATP produced. Flux sampling results from this scenario predicted a range of potential products (Fig. 6A), including ethanol, C2, H_2_, even-chain carboxylic acids, and odd-chain carboxylic acids. Overall, the amount of MCFAs predicted by iFermCell215 exceeded observed values, exposing a limitation of the single unit model.

In an attempt to better represent the compartmentalized nature of activities within mixed microbial communities, we developed the functional guild model iFermGuilds789. In iFermGuilds789, the metabolic activities of a mixed microbial community are partitioned among a number of functional guilds. This biological division of labor negates the free exchange of reducing equivalents (e.g., NADH, ferredoxin) and of many pathway intermediates (e.g., acetyl-CoA) between community members, ultimately constraining how individual organisms maintain redox balance, which can potentially impact ATP and product yields. For instance, the single unit model (iFermCell215) cannot predict the production of lactate via an NADH-dependent dehydrogenase and reconsumption of lactate via an electron-confurcating dehydrogenase, activities that may happen in a mixed microbial community that includes lactate producers and lactate consumers. The iFermGuilds789 model contains fermentation pathways in six guilds (Fig. 2), with each guild including predictions on transport reactions to simulate uptake and release of intermediate metabolites by the most abundant members of the community as predicted from previous metagenomics analyses (25).

To simulate the activities in the MCFA-producing bioreactor, we used a version of iFermGuilds789 with only four guilds, which represented the most abundant community members in the bioreactor as determined by metagenome sequencing (25). These guilds included the sugar-elongating “*Candidatus* Weimeria bifida” (SEOs), lactate-elongating “*Candidatus* Pseudoramibacter fermentans” (LEOs) (28), five sugar-fermenting *Lactobacillus* species (SFOs), and three hydrogenic sugar-fermenting *Coriobacteriaceae* species (HSFs) (25). We constrained the abundance of each guild in iFermGuilds789 according to the relative abundance of DNA for each guild in the bioreactor. Guilds were assumed to have all pathways associated with their guild (Fig. 2), and we required each guild to have the same growth rate of 0.0069 h^−1^. The results of this iFermGuilds789 simulation (Fig. 6B) predicted a narrower range of products than iFermCell215 (Fig. 6A), with even- and odd-chain fatty acid products predicted. With these parameters, the iFermGuilds789 model overpredicted the production of C2 and C8 while underpredicting C4 and C6 production.

In an additional iFermGuilds789 simulation, we restricted pathways in guild members based on previous metatranscriptomic analyses (25). SEOs were constrained to use glucose, xylose, and glycerol; SFOs were restricted to consuming glucose, xylose, xylans, glucans, and glycerol; HSFs were constrained to consume only glucans, glucose, and glycerol; and LEOs were restricted to consuming only lactate and glycerol. Further, SEOs and LEOs were constrained to not produce odd-chain volatile fatty acids (propionate, pentanoate, and heptanoate). LEOs were further constrained to not use acetate kinase, which is absent from the genome of “*Candidatus* Pseudoramibacter fermentans” (28). This iFermGuilds789 simulation predicted ranges of ethanol, lactate, C2, and C6 that contained the experimentally observed values (Fig. 6C). With these parameters, C8 and H_2_ production was overpredicted by iFermGuilds789, while C4 production was underpredicted.

One potential explanation for the differences in the various iFermGuilds789 predictions may be the specificity of chain-elongating enzymes for producing products of defined chain lengths. This could include any of the enzymes catalyzing the four steps of reverse β-oxidation itself or the terminal enzymes that cleave the final products of reverse β-oxidation from CoA. We tested this by constraining the flux through CoAT and acyl-CoA hydrolase reactions for both SEOs and LEOs according to carbon-chain length. When constraining both CoAT and acyl-CoA hydrolase in both SEOs and LEOs to 0.02 mmol g dry cell weight (DCW)^−1^ h^−1^ for C8 and 1 mmol gDCW^−1^ h^−1^ for C6, iFermGuilds789 modeling predictions aligned much better with community performance (Fig. 6D). This suggests that reverse β-oxidation and/or terminal enzymes in this pathway have higher affinity for C4 and C6 than for C8 in this specific microbial community.

Taken together, initial predictions from iFermCell215 (Fig. 6A) and iFermGuilds789 (Fig. 6B) did not align with observed bioreactor performance. Predictions of iFermGuilds789 improved by incorporating knowledge of specific reactions within each guild (Fig. 6C). Further, the best alignment between observations and model predictions resulted from constraining the fluxes related to individual products with iFermGuilds789 (Fig. 6D). This suggests that accurate predictions of MCFA production may require incorporation of enzyme affinity and additional characterization of enzymes from chain-elongating species to improve modeling predictions.

### Assessing interspecies metabolite flux using iFermGuilds789.

Our previous analyses predicted that microbes within the MCFA-producing microbial community perform different functions, compete for resources, and participate in interspecies transfer of metabolites that allows for conversion of lignocellulosic biorefinery residues to SCFAs and MCFAs (25). We used iFermGuilds789 to predict the function of each guild in the transformation of substrates as well as the production and transfer of intermediate metabolites between microbes. In this case, unlike previous simulations with iFermGuilds789, we did not constrain the growth rate (Fig. 6B to D) or fluxes for MCFA production (Fig. 6D). Instead, we constrained iFermGuilds789 to accumulate extracellular products at the rates observed in the MCFA-producing bioreactor (Table S2), constrained the reactions within each guild as described above, and predicted fluxes using parsimonious flux balance analysis (pFBA), which calculates a single flux distribution when the model is constrained to the observed production rates. Under these conditions, iFermGuilds789 predicted a growth rate of 0.0051 h^−1^, which is 74% of the expected growth rate (0.0069 h^−1^). This growth rate corresponds to a retention time of 8 days (compared to the actual retention time of 6 days), and these differences may reflect difficulty in estimating the amount of metabolically active cells in the reactor or absence of unknown metabolic pathways that may increase the predicted growth rate.

**FIG 7:**
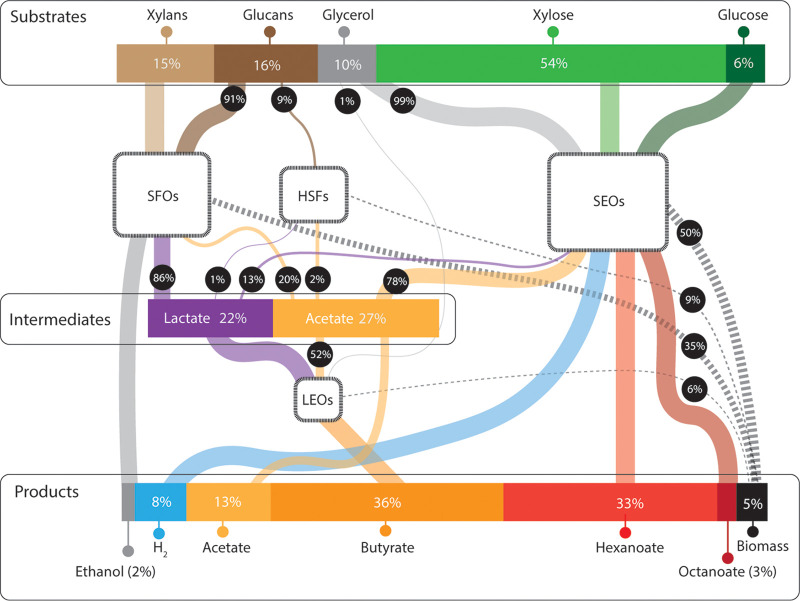
Predicted substrate flow through a mixed-culture fermentation producing a mixture of MCFAs from a complex feedstock using iFermGuilds789. Values were obtained using pFBA while constraining the products according to observed bioreactor performance. The relative abundance of each functional guild was assumed based on reactor DNA abundance. The relative amounts of eeq in the substrates, products, and intermediates are also provided. Line width indicates the relative amount of each substrate consumed and product created by each of the functional guilds. H_2_ was not measured, so for these simulations it was assumed that the difference between eeq in the substrate and eeq in the measured soluble and insoluble products represented H_2_ production. In this simulation, 22% of the eeq in conversion residue was predicted to be directed to lactate as an intermediate and 27% was predicted to be directed to acetate.

By constraining the substrates and products according to observed community behavior, iFermGuilds789 predicted that SFOs and HSFs consumed xylans and glucans, while SEOs consumed xylose, glucose, and 99% of the glycerol (Fig. 7). SFOs were predicted to produce ethanol, lactate, and C2, while HSFs were predicted to produce a small amount of the lactate and C2. SEOs are also predicted to produce lactate and C2. In total, 22% of the eeq in the feedstock are predicted by this instance of iFermGuilds789 to be converted to lactate and 27% are predicted to be converted to C2. All of the lactate and 52% of the C2 are predicted to be consumed by LEOs. LEOs are predicted to produce all of the C4, and the C4 is not predicted to be consumed by other guilds. SEOs are predicted to produce all of the C6 and C8 along with all of the H_2_ from carbohydrates. SEOs are further predicted to have a flux for C6 production of 0.46 mmol gDCW^−1^ h^−1^ and C8 production of 0.033 mmol gDCW^−1^ h^−1^ (Data Set S5), both of which are lower than the maximum flux constraints applied previously (Fig. 6D).

### Predicted routes of sugar and lactate elongation.

**FIG 8:**
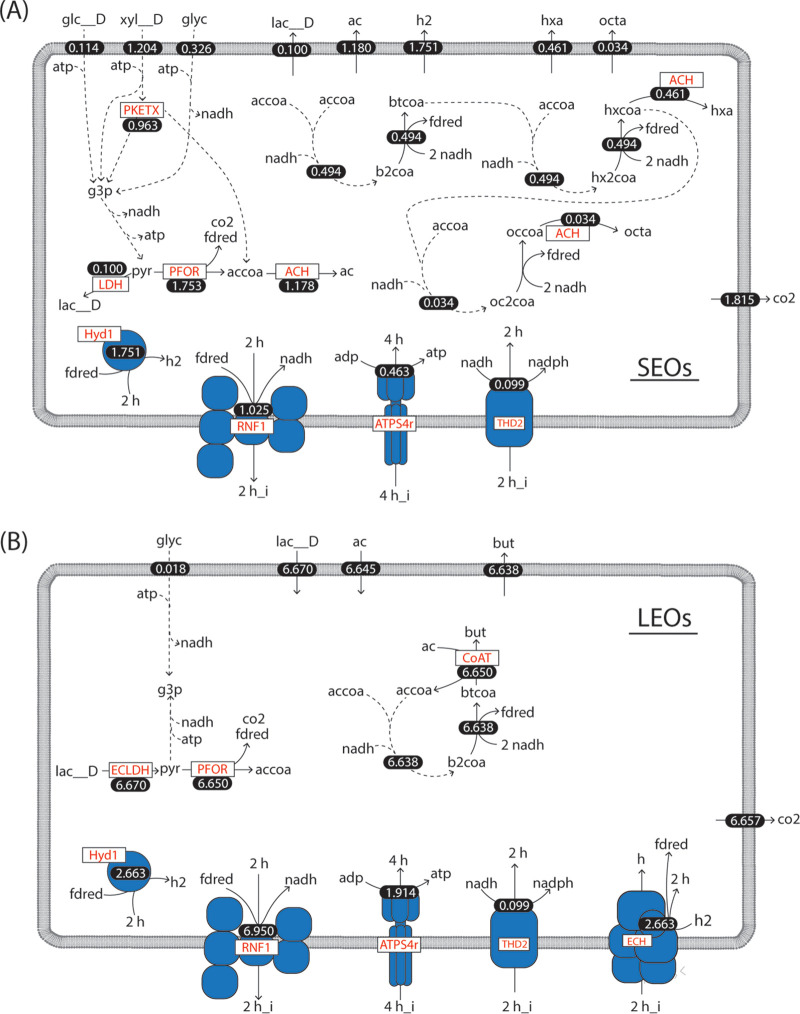
Predicted chain elongation metabolism by SEOs and LEOs using iFermGuilds789. Predicted flux values are shown as white numbers and have units of mmol gDCW^−1^ h^−1^. (A) SEOs are predicted to produce all of the hexanoate (hxa) and octanoate (octa) in the community and use an acyl-CoA hydrolase (ACH) reaction as the terminal step of reverse β-oxidation. (B) LEOs are predicted to produce all of the butyrate in the community while using a CoA transferase (CoAT). LEOs are predicted to recycle all of the H_2_ that they produce via an energy-conserving hydrogenase. Metabolites are defined in Data Set S3.

The above prediction that essentially all MCFAs were produced by SEOs consuming xylose, glycerol, and glucose, while LEOs produced C4 from lactate and C2, was different than the predictions made earlier based on transcriptomic analyses alone (25). To gain further insights into SEO and LEO metabolism, we investigated the specific reactions predicted for both guilds by iFermGuilds789. SEOs are predicted to produce pyruvate via glycolysis. Some pyruvate is predicted to be converted to lactate, while most is converted to acetyl-CoA via pyruvate flavodoxin oxidoreductase (PFOR, Fig. 8A). iFermGuilds789 also predicted that acetyl-CoA is derived from the phosphoketolase pathway (PKETX, Fig. 8A). Acetyl-CoA is predicted to be directed to reverse β-oxidation, but a portion is also predicted to be hydrolyzed by an acyl-CoA hydrolase. Acyl-CoA hydrolase enzymes are also predicted as terminal enzymes of reverse β-oxidation rather than energy-conserving CoA transferase enzymes (CoAT). SEOs are further predicted to use Hyd1 for hydrogen production, to generate IMF via RNF1, and to produce ATP via ATP synthase (ATPS4r) (Fig. 8A).

LEOs are predicted to convert lactate to pyruvate via an electron-confurcating lactate dehydrogenase (ECLDH, Fig. 8B), and pyruvate is predicted to form acetyl-CoA via PFOR. All of the acetyl-CoA is predicted to undergo reverse β-oxidation, and C2 is incorporated by a CoAT that transfers the CoA from butyryl-CoA to acetyl-CoA (Fig. 8B). LEOs are predicted to consume equimolar concentrations of C2 and lactate, which was previously predicted to favor C4 production (Fig. 4). Further, LEOs are not predicted to produce H_2_. iFermGuilds789 predicted that H_2_ produced by Hyd1 is reconsumed via a proton-translocating energy-conserving hydrogenase that produces reduced ferredoxin (ECH, Fig. 8B). This ferredoxin augments that produced by PFOR and is predicted to be used for lactate consumption by ECLDH and the RNF complex. The use of an energy-conserving hydrogenase for ferredoxin production from H_2_ has been demonstrated previously (29).

While both SEOs and LEOs are predicted by iFermGuilds789 to use reverse β-oxidation to elongate acetyl-CoA, the energy-conserving and redox-balancing reactions needed to support this process are predicted to differ. While SEOs are predicted to use an acyl-CoA hydrolase as the terminal enzyme of reverse β-oxidation, LEOs are predicted to use a CoAT which conserves energy by activating C2 to acetyl-CoA, which would otherwise require ATP. In the previous metatranscriptomic analyses of these community members (25, 28), we found that both SEOs and LEOs expressed genes for both acyl-CoA hydrolases and CoAT. The specificity of these predicted chain-elongating enzymes for different substrates, however, is not known. The iFermGuilds789 model predictions reinforce the potential importance of terminal enzymes in reverse β-oxidation in determining the final product length; specifically, the model predicts that all C4 is produced using CoAT and that all MCFAs are produced using acyl-CoA hydrolase.

Furthermore, SEOs are predicted by iFermGuilds789 to maintain redox balance by producing H_2_ with Hyd1, while LEOs are predicted to recycle all H_2_ internally using both Hyd1 and ECH. Previous metatranscriptomic analyses showed that both LEOs and SEOs expressed genes for both Hyd1 and ECH (25). This agrees with iFermGuilds789 predictions for LEOs, but not for SEOs, which are predicted to use only Hyd1. When constraining iFermGuilds789 to require SEOs to use ECH, there are no changes in interspecies metabolite transfer (Data Set S5), but SEOs are predicted to generate additional ATP via ATP synthase while reducing flux through other energy-conserving pathways to achieve the same growth rate (Data Set S5). These findings suggest that SEOs and LEOs have multiple methods for conserving energy and maintaining redox balance and that this metabolic flexibility can maintain the same overall transformation of complex substrates to products. This metabolic flexibility may reflect ecological advantages for fermenting organisms but also challenges for the accurate predictions of MCFA production, which may strongly depend on the specific organisms present in the microbial community.

### Implications of modeling results.

The predictions of our models generated several hypotheses that should be explored with further analyses. First, the single unit iFermCell215 predicted production of MCFAs from ethanol as a sole source of electrons and carbon (Fig. 4A to D), and this is supported by recent experiments with mixed cultures (21). More work is needed to determine bioreactor conditions that promote elongation of ethanol as a sole substrate or with low C2:EtOH ratios. The iFermCell215 simulations predict a threshold of 0.56 mol C2 per mol ethanol below which C8 production benefits from C2 coutilization and above which C8 production starts to decrease and to be replaced by production of shorter acids. This shift in product profile was predicted by iFermCell215 to be associated with reversal in the direction of enzymatic activities related to energy-conserving metabolism, an observation that still lacks experimental verification.

Second, even though lactate is a known substrate for chain elongation (9), iFermGuilds789 simulation results suggest that, in this mixed community, lactate may be converted to C4 instead of C6 or C8 (Fig. 7). Although at lower C2-to-lactate ratios iFermCells215 simulations predicted that this ratio was not as influential on determining the product distribution as the C2:EtOH ratio, the model also predicted that excess C2 could result in C4 production instead of C6 or C8 production from both intermediate metabolites (Fig. 4). The iFermGuilds789 simulation predicted coutilization of C2 and lactate at a molar ratio of 1.0 (Fig. 8B), and according to the iFermCell215 simulations (Fig. 4), this is already an excess C2 condition predicted to yield C4 as the main fermentation product. These results have the important implication that efforts directed to construct synthetic communities or control self-assembled communities for production of MCFA should focus on minimizing C2 production and accumulation.

Third, iFermCell215 modeling results suggest that controlling product formation from lactate may not be possible by controlling C2-to-lactate ratios (Fig. 4E to G), whereas C2:EtOH ratios suggest very specific targets to maximize C8 production (Fig. 4A to D). This suggests that for MCFA production from complex organic substrates, in addition to targeting the reduction of C2 production, efforts should also be directed to creating conditions under which ethanol instead of lactate is the key intermediate metabolite for MCFA production. However, this may not be achievable in self-assembled communities, and since simulations predict that the products of lactate fermentation are dependent on specific metabolic features of LEOs (Fig. S2), it is important to continue the discovery of the natural diversity of lactate fermentation metabolisms, which eventually could help identify preferred LEOs for the development of novel strategies to promote MCFA production when lactate is the key intermediate metabolite.

Fourth, iFermGuilds789 predicts that enzyme affinity plays a major role in determining the final products of reverse β-oxidation and mixed-culture fermentation (Fig. 6D). Additional work is needed to assess the affinity of reverse β-oxidation, acyl-CoA hydrolase, and CoA transferase enzymes for different chain lengths to identify ideal microorganisms or to engineer microorganisms to improve MCFA production. Past studies have identified and engineered acyl-ACP thioesterase enzymes (the terminal enzyme of fatty acid synthesis via the fatty acid biosynthesis pathway) with high affinity for C8 (30–32). Similar studies should be performed with acyl-CoA hydrolase and acyl-CoA transferase enzymes to characterize and improve the terminal enzymes of reverse β-oxidation.

Since many microbes in natural and self-assembled fermentation communities remain uncultured, our knowledge and ability to predict important metabolic networks rely largely on information from metagenomics, metatranscriptomics, and metabolomics analyses to build the functional activities in each guild (25, 33). We show in this work that metabolic modeling offers additional insight that is not gained by these analyses but that is critical in the evaluation of the microbial communities and in developing a hypothesis of how to direct metabolism toward a specific set of fermentation products. From prior analyses (25), we anticipated that both SEOs and LEOs participated in MCFA production, but the iFermGuilds789 simulation of interspecies metabolite flux (Fig. 7) predicted that to achieve the observed growth rate of the microbial community, C6 and C8 were produced solely by SEOs, while LEOs were responsible for production of C4. This difference between predictions based on metatranscriptomic and simulation analyses can be tracked to the importance of coutilization of fermentation products (i.e., C2 and lactate), which is easier to explore using metabolic models.

iFermCell215 and iFermGuilds789 are examples of first-generation predictive models of a mixed-culture fermentation. Their utility can be improved by obtaining additional knowledge of metabolic pathways involved in fermentation through studying individual microbes within each functional guild. Further effort is needed to simulate poorly appreciated conditions that shape community structure, such as the toxicity of fermentation products on the specific guilds or the contribution(s) of low-abundance microbes to the overall activity of the mixed-culture fermentation. Further, the objective function to maximize biomass growth may not always represent the true objective of a fermenting microbial community. While there is no direct evidence that polymer storage is occurring in these organisms, other undescribed traits that enable resiliency may be present. Additionally, while most studies investigating the minimum required free energy release have found values close to 50 kJ per mol ATP (34), ATP production may be possible with less free energy release (35). Lastly, computational methods to predict the abundance of specific guilds, rather than relying on relative abundances as a model input, are needed. Efforts to assemble such models have begun for the human gut microbiome (36, 37), and as these approaches are applied to mixed microbial fermentations, their benefits to the production of MCFA and other chemicals of societal importance will continue to improve.

## MATERIALS AND METHODS

Metabolic networks of mixed-culture fermentation that contain reactions that occur during anaerobic metabolism of a previously described lignocellulosic biorefinery residue (25) were assembled. The first network, iFermCell215, contains 215 reactions and 154 metabolites, models mixed-culture fermentation as a single unit, and expands the metabolic pathways and substrate range of previously published mixed-culture fermentation networks (11–13). iFermCell215 describes metabolism of exogenous substrates including glucans, glucose, xylans, xylose, and glycerol, along with lactate, ethanol, acetate, CO_2_, and H_2_, which are potential intermediates in this mixed-culture fermentation (Fig. 1). The code for iFermCell215 is provided in Data Set S1 in the supplemental material.

10.1128/mSystems.00755-20.6 DATA SET S1 Calculations for obtaining the simplified biomass equation. Download Data Set S1, TXT file, 0.1 MB. Copyright © 2020 Scarborough et al. 2020 Scarborough et al. This content is distributed under the terms of the Creative Commons Attribution 4.0 International license.

10.1128/mSystems.00755-20.7 DATA SET S2 iFermCell215 metabolic model in .py format. Download Data Set S2, TXT file, 0.6 MB. Copyright © 2020 Scarborough et al. 2020 Scarborough et al. This content is distributed under the terms of the Creative Commons Attribution 4.0 International license.

10.1128/mSystems.00755-20.8 DATA SET S3 iFermGuilds789 metabolic model in .py format. Download Data Set S3, XLSX file, 0.2 MB. Copyright © 2020 Scarborough et al. 2020 Scarborough et al. This content is distributed under the terms of the Creative Commons Attribution 4.0 International license.

10.1128/mSystems.00755-20.9 DATA SET S4 Lists of reactions and metabolites for both metabolic models. Download Data Set S4, XLSX file, 0.1 MB. Copyright © 2020 Scarborough et al. 2020 Scarborough et al. This content is distributed under the terms of the Creative Commons Attribution 4.0 International license.

10.1128/mSystems.00755-20.10 DATA SET S5 Flux predictions from metabolic modeling results used in this study. Download Data Set S5, XLSX file, 11.8 MB. Copyright © 2020 Scarborough et al. 2020 Scarborough et al. This content is distributed under the terms of the Creative Commons Attribution 4.0 International license.

The second network, iFermGuilds789, represents activities of different guilds (i.e., groups of organisms with similar functions) within the microbial community. The iFermGuilds789 network describes the same reactions and metabolites as in the iFermCell215 model, but these are separated into six functional guilds with each guild representing a subset of compartmentalized reactions (Fig. 2). iFermGuilds789 also includes additional transport reactions that simulate metabolite exchange among guilds. Combining the six guilds, the iFermGuilds789 network includes 789 reactions and 579 metabolites. The guilds in the iFermGuilds789 model represent previously described organisms that perform fatty acyl chain elongation from sugars (SEOs), sugar-fermenting organisms that produce lactate, acetate, and ethanol as fermentation products (SFO), hydrogen-producing sugar fermenters (HSFs), organisms that perform fatty acyl chain elongation from lactate (LEOs) or ethanol (EEOs), and homoacetogenic organisms (HAOs). These guilds were selected based on organisms that have been found in MCFA-producing bioreactors (25, 33) or suggested to play a role in MCFA production (13). Examples of organisms falling within each functional guild are described in Text S1. The code for iFermGuilds789 is provided in Data Set S2.

10.1128/mSystems.00755-20.1 TEXT S1 Additional descriptions of representative organisms in each guild, formation of the biomass equation, transport constraints, and the predicted impacts of reaction knockouts on lactate consumption. Download Text S1, DOCX file, 0.1 MB. Copyright © 2020 Scarborough et al. 2020 Scarborough et al. This content is distributed under the terms of the Creative Commons Attribution 4.0 International license.

When constructing each metabolic network, reactions were added to capture the exchange of substrates and products with the extracellular space (38). In addition to intracellular (denoted by “_c” in the metabolic models) and extracellular (“_e”) compartments, a compartment for ion-motive force generation was included (denoted by “_i” in the metabolic models). Where possible, standard metabolite and reaction abbreviations from the Biochemical, Genetic, and Genomic (BiGG) knowledgebase were used (39). All reactions were balanced for mass and charge assuming an intracellular pH of 7.0 with the dominant ionic form of all metabolites obtained from the BiGG database (39).

Since many members of this mixed microbial community are *Firmicutes*, simplified biomass equations for both models were created based on the biomass composition of the well-studied Gram-positive bacterium Bacillus subtilis (40–42). We simplified all of the biosynthetic pathways for production of biomass components to capture 16 precursor compounds produced by the models. The precursor compounds included α-ketoglutarate, oxaloacetate, glucose-6-phosphate, glucose-3-phosphate, 3-phosphoglycerate, pyruvate, phosphoenolpyruvate, acetyl-CoA, erythrose-4-phosphate, ribulose-5-phosphate, ATP, NADPH, NAD^+^, ammonium, sulfate, and H_2_O. Additional information and calculations for the biomass equation are provided in Text S1 and Data Set S3. Both metabolic models (iFermCell215 and iFermGuilds789) and instructions for their use are also available at https://github.com/mscarbor/Mixed-Culture-Fermentation-Models.

The production capabilities of both metabolic networks were simulated using standard constraint-based methods (43). Given a defined set of substrate fluxes entering the system from the outside environment (the _e compartment), these methods predict the steady-state flux distribution out of the cell and through the metabolic networks created by individual reactions, subject to physiochemical constraints. In this work, we used flux sampling (44) and parsimonious flux balance analysis (pFBA) (45). Flux sampling was performed using artificial centering hit and run (ACHR) (44) using 101 simulations and randomly generated seeds. A solution with pFBA provides a flux distribution by minimizing the sum of all fluxes predicted by the model (45). When modeling bioreactors with the presence of solute gradients across the cell membrane, we considered the ATP required for transport as described in Text S1. The objective function for all modeling scenarios was to maximize biomass production. When simulating the iFermGuilds789 network for a steady-state condition, we required all individual guilds to achieve the same growth rate (h^−1^). All simulations were performed in Python 3.7 using the COBRApy package (v 0.18.1) (46).

Throughout this work, we present results in terms of electron equivalents (eeq), which report the number of electrons potentially transferred to an electron acceptor if the compound is completely oxidized (using factors provided in Table S1 to convert from molar concentrations to eeq concentrations). For each scenario, we calculated the overall reaction thermodynamics using the predicted stoichiometry of the model and the standard free energy of formation (Δ*G*_f_^0^) of metabolites (Table S1) obtained from KBase (47). Unless otherwise noted, we ensured that at least 50 kJ was available per ATP produced with standard concentrations (all molar concentrations = 1 M and partial pressures = 1 atm) at a temperature of 35°C and at neutral pH (15, 48).

10.1128/mSystems.00755-20.4 TABLE S1 Summary of metabolites that are consumed or produced in the metabolic models used in this study. Chemical formulas are provided for the predominant form at a pH of 7.0. Download Table S1, DOCX file, 0.01 MB. Copyright © 2020 Scarborough et al. 2020 Scarborough et al. This content is distributed under the terms of the Creative Commons Attribution 4.0 International license.

10.1128/mSystems.00755-20.5 TABLE S2 Calculated electron equivalents (eeq) contained in substrates and products from a mixed-culture fermentation of lignocellulosic biorefinery residue. Substrates represent the eeq consumed by the microbial community, and products represent the millielectron equivalents (meeq) of the measured products. We assumed that the complex carbohydrates remaining in conversion residue were comprised of 50% xylans and 50% glucans, by mass based on published compositions of this material (5). The bioreactor performance data are from day 96 of reactor operations described in the work of Scarborough et al., 2018 (25). Download Table S2, DOCX file, 0.01 MB. Copyright © 2020 Scarborough et al. 2020 Scarborough et al. This content is distributed under the terms of the Creative Commons Attribution 4.0 International license.

## References

[1] McGovern PE, Zhang J, Tang J, Zhang Z, Hall GR, Moreau RA, Nunez A, Butrym ED, Richards MP, Wang CS, Cheng G, Zhao Z, Wang C. 2004. Fermented beverages of pre- and proto-historic China. Proc Natl Acad Sci U S A 101:17593–17598. doi:10.1073/pnas.0407921102. 15590771 PMC539767

[2] Bond T, Templeton MR. 2011. History and future of domestic biogas plants in the developing world. Energy Sustain Dev 15:347–354. doi:10.1016/j.esd.2011.09.003.

[3] Angenent LT, Richter H, Buckel W, Spirito CM, Steinbusch KJJ, Plugge CM, Strik D, Grootscholten TIM, Buisman CJN, Hamelers HVM. 2016. Chain elongation with reactor microbiomes: open-culture biotechnology to produce biochemicals. Environ Sci Technol 50:2796–2810. doi:10.1021/acs.est.5b04847. 26854969

[4] Sarria S, Kruyer NS, Peralta-Yahya P. 2017. Microbial synthesis of medium-chain chemicals from renewables. Nat Biotechnol 35:1158–1166. doi:10.1038/nbt.4022. 29220020

[5] Scarborough MJ, Lynch G, Dickson M, McGee M, Donohue TJ, Noguera DR. 2018. Increasing the economic value of lignocellulosic stillage through medium-chain fatty acid production. Biotechnol Biofuels 11:200. doi:10.1186/s13068-018-1193-x. 30034526 PMC6052542

[6] Nelson R, Peterson D, Karp E, Beckham G, Salvachúa D. 2017. Mixed carboxylic acid production by Megasphaera elsdenii from glucose and lignocellulosic hydrolysate. Fermentation 3:10. doi:10.3390/fermentation3010010.

[7] Kucek LA, Xu JJ, Nguyen M, Angenent LT. 2016. Waste conversion into n-caprylate and n-caproate: resource recovery from wine lees using anaerobic reactor microbiomes and in-line extraction. Front Microbiol 7:1892. doi:10.3389/fmicb.2016.01892. 27933053 PMC5121217

[8] Zhu X, Zhou Y, Wang Y, Wu T, Li X, Li D, Tao Y. 2017. Production of high-concentration n-caproic acid from lactate through fermentation using a newly isolated Ruminococcaceae bacterium CPB6. Biotechnol Biofuels 10:102. doi:10.1186/s13068-017-0788-y. 28439295 PMC5399333

[9] Kucek LA, Nguyen M, Angenent LT. 2016. Conversion of L-lactate into n-caproate by a continuously fed reactor microbiome. Water Res 93:163–171. doi:10.1016/j.watres.2016.02.018. 26905795

[10] Coma M, Vilchez-Vargas R, Roume H, Jauregui R, Pieper DH, Rabaey K. 2016. Product diversity linked to substrate usage in chain elongation by mixed-culture fermentation. Environ Sci Technol 50:6467–6476. doi:10.1021/acs.est.5b06021. 27162101

[11] Rodriguez J, Kleerebezem R, Lema JM, van Loosdrecht MCM. 2006. Modeling product formation in anaerobic mixed culture fermentations. Biotechnol Bioeng 93:592–606. doi:10.1002/bit.20765. 16273553

[12] Zhang F, Zhang Y, Chen M, van Loosdrecht MCM, Zeng RJ. 2013. A modified metabolic model for mixed culture fermentation with energy conserving electron bifurcation reaction and metabolite transport energy. Biotechnol Bioeng 110:1884–1894. doi:10.1002/bit.24855. 23381671

[13] Regueira A, González-Cabaleiro R, Ofiţeru ID, Rodríguez J, Lema JM. 2018. Electron bifurcation mechanism and homoacetogenesis explain products yields in mixed culture anaerobic fermentations. Water Res 141:349–356. doi:10.1016/j.watres.2018.05.013. 29804021

[14] Regueira A, Lema JM, Carballa M, Mauricio-Iglesias M. 2020. Metabolic modeling for predicting VFA production from protein-rich substrates by mixed-culture fermentation. Biotechnol Bioeng 117:73–84. doi:10.1002/bit.27177. 31544960

[15] Thauer RK, Jungermann K, Decker K. 1977. Energy conservation in chemotrophic anaerobic bacteria. Bacteriol Rev 41:100–180. doi:10.1128/MMBR.41.1.100-180.1977. 860983 PMC413997

[16] Han WH, He PJ, Shao LM, Lu F. 2019. Road to full bioconversion of biowaste to biochemicals centering on chain elongation: a mini review. J Environ Sci (China) 86:50–64. doi:10.1016/j.jes.2019.05.018. 31787190

[17] Spirito CM, Richter H, Rabaey K, Stams AJ, Angenent LT. 2014. Chain elongation in anaerobic reactor microbiomes to recover resources from waste. Curr Opin Biotechnol 27:115–122. doi:10.1016/j.copbio.2014.01.003. 24487179

[18] Schoberth S, Gottschalk G. 1969. Considerations on the energy metabolism of Clostridium kluyveri. Arch Mikrobiol 65:318–328. doi:10.1007/BF00412211. 5384695

[19] Thauer RK, Jungermann K, Henninger H, Wenning J, Decker K. 1968. The energy metabolism of Clostridium kluyveri. Eur J Biochem 4:173–180. doi:10.1111/j.1432-1033.1968.tb00189.x. 5655494

[20] Yin Y, Zhang Y, Karakashev DB, Wang J, Angelidaki I. 2017. Biological caproate production by Clostridium kluyveri from ethanol and acetate as carbon sources. Bioresour Technol 241:638–644. doi:10.1016/j.biortech.2017.05.184. 28605728

[21] Spirito CM, Marzilli AM, Angenent LT. 2018. Higher substrate ratios of ethanol to acetate steered chain elongation toward n-caprylate in a bioreactor with product extraction. Environ Sci Technol 52:13438–13447. doi:10.1021/acs.est.8b03856. 30335369

[22] Roghair M, Hoogstad T, Strik D, Plugge CM, Timmers PHA, Weusthuis RA, Bruins ME, Buisman CJN. 2018. Controlling ethanol use in chain elongation by CO2 loading rate. Environ Sci Technol 52:1496–1505. doi:10.1021/acs.est.7b04904. 29304274 PMC5997387

[23] Weimer PJ, Moen GN. 2013. Quantitative analysis of growth and volatile fatty acid production by the anaerobic ruminal bacterium Megasphaera elsdenii T81. Appl Microbiol Biotechnol 97:4075–4081. doi:10.1007/s00253-012-4645-4. 23271673

[24] Zhu X, Tao Y, Liang C, Li X, Wei N, Zhang W, Zhou Y, Yang Y, Bo T. 2015. The synthesis of n-caproate from lactate: a new efficient process for medium-chain carboxylates production. Sci Rep 5:14360. doi:10.1038/srep14360. 26403516 PMC4585908

[25] Scarborough MJ, Lawson CE, Hamilton JJ, Donohue TJ, Noguera DR. 2018. Metatranscriptomic and thermodynamic insights into medium-chain fatty acid production using an anaerobic microbiome. mSystems 3:e00221-18. doi:10.1128/mSystems.00221-18. 30505946 PMC6247018

[26] Andersen SJ, De Groof V, Khor WC, Roume H, Props R, Coma M, Rabaey K. 2017. A Clostridium group IV species dominates and suppresses a mixed culture fermentation by tolerance to medium chain fatty acids products. Front Bioeng Biotechnol 5:8. doi:10.3389/fbioe.2017.00008. 28265558 PMC5316547

[27] Seedorf H, Fricke WF, Veith B, Bruggemann H, Liesegang H, Strittmatter A, Miethke M, Buckel W, Hinderberger J, Li F, Hagemeier C, Thauer RK, Gottschalk G. 2008. The genome of Clostridium kluyveri, a strict anaerobe with unique metabolic features. Proc Natl Acad Sci U S A 105:2128–2133. doi:10.1073/pnas.0711093105. 18218779 PMC2542871

[28] Scarborough MJ, Myers KS, Donohue TJ, Noguera DR. 2019. Medium-chain fatty acid synthesis by “Candidatus Weimeria bifida” gen. nov., sp. nov., and “Candidatus Pseudoramibacter fermentans” sp. nov. Appl Environ Microbiol 86:e02242-19. doi:10.1128/AEM.02242-19. PMC6974650 31704684

[29] Hedderich R, Forzi L. 2005. Energy-converting [NiFe] hydrogenases: more than just H-2 activation. J Mol Microbiol Biotechnol 10:92–104. doi:10.1159/000091557. 16645307

[30] Sarria S, Bartholow TG, Verga A, Burkart MD, Peralta-Yahya P. 2018. Matching protein interfaces for improved medium-chain fatty acid production. ACS Synth Biol 7:1179–1187. doi:10.1021/acssynbio.7b00334. 29722970 PMC6296256

[31] Hernandez Lozada NJ, Lai RY, Simmons TR, Thomas KA, Chowdhury R, Maranas CD, Pfleger BF. 2018. Highly active C8-acyl-ACP thioesterase variant isolated by a synthetic selection strategy. ACS Synth Biol 7:2205–2215. doi:10.1021/acssynbio.8b00215. 30064208 PMC6553638

[32] Deng X, Chen L, Hei M, Liu T, Feng Y, Yang GY. 2020. Structure-guided reshaping of the acyl binding pocket of ′TesA thioesterase enhances octanoic acid production in E. coli. Metab Eng 61:24–32. doi:10.1016/j.ymben.2020.04.010. 32339761

[33] Han W, He P, Shao L, Lu F. 2018. Metabolic interactions of a chain elongation microbiome. Appl Environ Microbiol 84:e01614-18. doi:10.1128/AEM.01614-18. 30217839 PMC6210121

[34] Muller V, Hess V. 2017. The minimum biological energy quantum. Front Microbiol 8:2019. doi:10.3389/fmicb.2017.02019. 29123504 PMC5662883

[35] Liu Y, Gu M, Yin Q, Du J, Wu G. 2020. Thermodynamic analysis of direct interspecies electron transfer in syntrophic methanogenesis based on the optimized energy distribution. Bioresour Technol 297:122345. doi:10.1016/j.biortech.2019.122345. 31706892

[36] Zomorrodi AR, Maranas CD. 2012. OptCom: a multi-level optimization framework for the metabolic modeling and analysis of microbial communities. PLoS Comput Biol 8:e1002363. doi:10.1371/journal.pcbi.1002363. 22319433 PMC3271020

[37] Kumar M, Ji B, Zengler K, Nielsen J. 2019. Modelling approaches for studying the microbiome. Nat Microbiol 4:1253–1267. doi:10.1038/s41564-019-0491-9. 31337891

[38] Covert MW, Schilling CH, Famili I, Edwards JS, Goryanin II, Selkov E, Palsson BO. 2001. Metabolic modeling of microbial strains in silico. Trends Biochem Sci 26:179–186. doi:10.1016/s0968-0004(00)01754-0. 11246024

[39] King ZA, Lu J, Drager A, Miller P, Federowicz S, Lerman JA, Ebrahim A, Palsson BO, Lewis NE. 2016. BiGG Models: a platform for integrating, standardizing and sharing genome-scale models. Nucleic Acids Res 44:D515–D522. doi:10.1093/nar/gkv1049. 26476456 PMC4702785

[40] Henry CS, Zinner JF, Cohoon MP, Stevens RL. 2009. iBsu1103: a new genome-scale metabolic model of Bacillus subtilis based on SEED annotations. Genome Biol 10:R69. doi:10.1186/gb-2009-10-6-r69. 19555510 PMC2718503

[41] Oh YK, Palsson BO, Park SM, Schilling CH, Mahadevan R. 2007. Genome-scale reconstruction of metabolic network in Bacillus subtilis based on high-throughput phenotyping and gene essentiality data. J Biol Chem 282:28791–28799. doi:10.1074/jbc.M703759200. 17573341

[42] Dauner M, Sauer U. 2001. Stoichiometric growth model for riboflavin-producing Bacillus subtilis. Biotechnol Bioeng 76:132–143. doi:10.1002/bit.1153. 11505383

[43] Bordbar A, Monk JM, King ZA, Palsson BO. 2014. Constraint-based models predict metabolic and associated cellular functions. Nat Rev Genet 15:107–120. doi:10.1038/nrg3643. 24430943

[44] Megchelenbrink W, Huynen M, Marchiori E. 2014. optGpSampler: an improved tool for uniformly sampling the solution-space of genome-scale metabolic networks. PLoS One 9:e86587. doi:10.1371/journal.pone.0086587. 24551039 PMC3925089

[45] Lewis NE, Hixson KK, Conrad TM, Lerman JA, Charusanti P, Polpitiya AD, Adkins JN, Schramm G, Purvine SO, Lopez-Ferrer D, Weitz KK, Eils R, Konig R, Smith RD, Palsson BO. 2010. Omic data from evolved E. coli are consistent with computed optimal growth from genome-scale models. Mol Syst Biol 6:390. doi:10.1038/msb.2010.47. 20664636 PMC2925526

[46] Ebrahim A, Lerman JA, Palsson BO, Hyduke DR. 2013. COBRApy: COnstraints-Based Reconstruction and Analysis for Python. BMC Syst Biol 7:74. doi:10.1186/1752-0509-7-74. 23927696 PMC3751080

[47] Arkin AP, Cottingham RW, Henry CS, Harris NL, Stevens RL, Maslov S, Dehal P, Ware D, Perez F, Canon S, Sneddon MW, Henderson ML, Riehl WJ, Murphy-Olson D, Chan SY, Kamimura RT, Kumari S, Drake MM, Brettin TS, Glass EM, Chivian D, Gunter D, Weston DJ, Allen BH, Baumohl J, Best AA, Bowen B, Brenner SE, Bun CC, Chandonia JM, Chia JM, Colasanti R, Conrad N, Davis JJ, Davison BH, DeJongh M, Devoid S, Dietrich E, Dubchak I, Edirisinghe JN, Fang G, Faria JP, Frybarger PM, Gerlach W, Gerstein M, Greiner A, Gurtowski J, Haun HL, He F, Jain R, Joachimiak MP, Keegan KP, Kondo S, Kumar V, Land ML, Meyer F, Mills M, Novichkov PS, Oh T, Olsen GJ, Olson R, Parrello B, Pasternak S, Pearson E, Poon SS, Price GA, Ramakrishnan S, Ranjan P, Ronald PC, Schatz MC, Seaver SMD, Shukla M, Sutormin RA, Syed MH, Thomason J, Tintle NL, Wang D, Xia F, Yoo H, Yoo S, Yu D. 2018. KBase: the United States Department of Energy systems biology knowledgebase. Nat Biotechnol 36:566–569. doi:10.1038/nbt.4163. 29979655 PMC6870991

[48] Alberty RA. 2003. Thermodynamics of biochemical reactions. John Wiley & Sons, Inc, Hoboken, NJ.

