# Supplementary figures and images for: Diagnosing and Predicting Mixed-Culture Fermentations with Unicellular and Guild-Based Metabolic Models
Source: mSystems. 2020 Sep 29;5(5):e00755-20. doi: 10.1128/mSystems.00755-20 (PMC7527139; doi:10.1128/mSystems.00755-20)

(A)

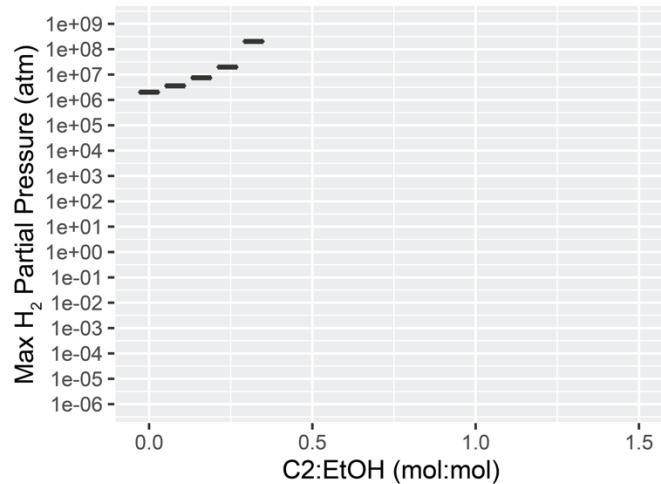

(B)

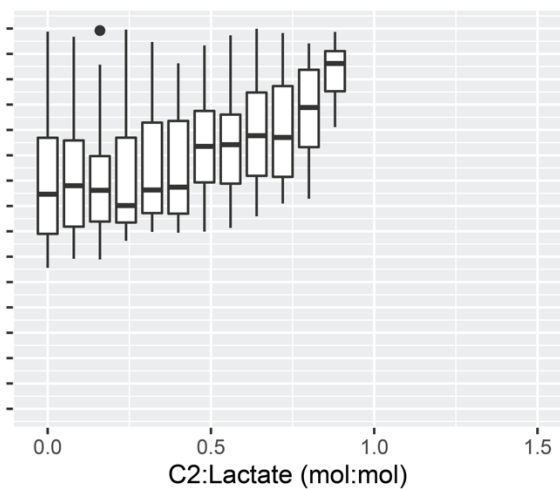

Supplement: FIG S1 [file mSystems.00755-20-sf001.pdf]

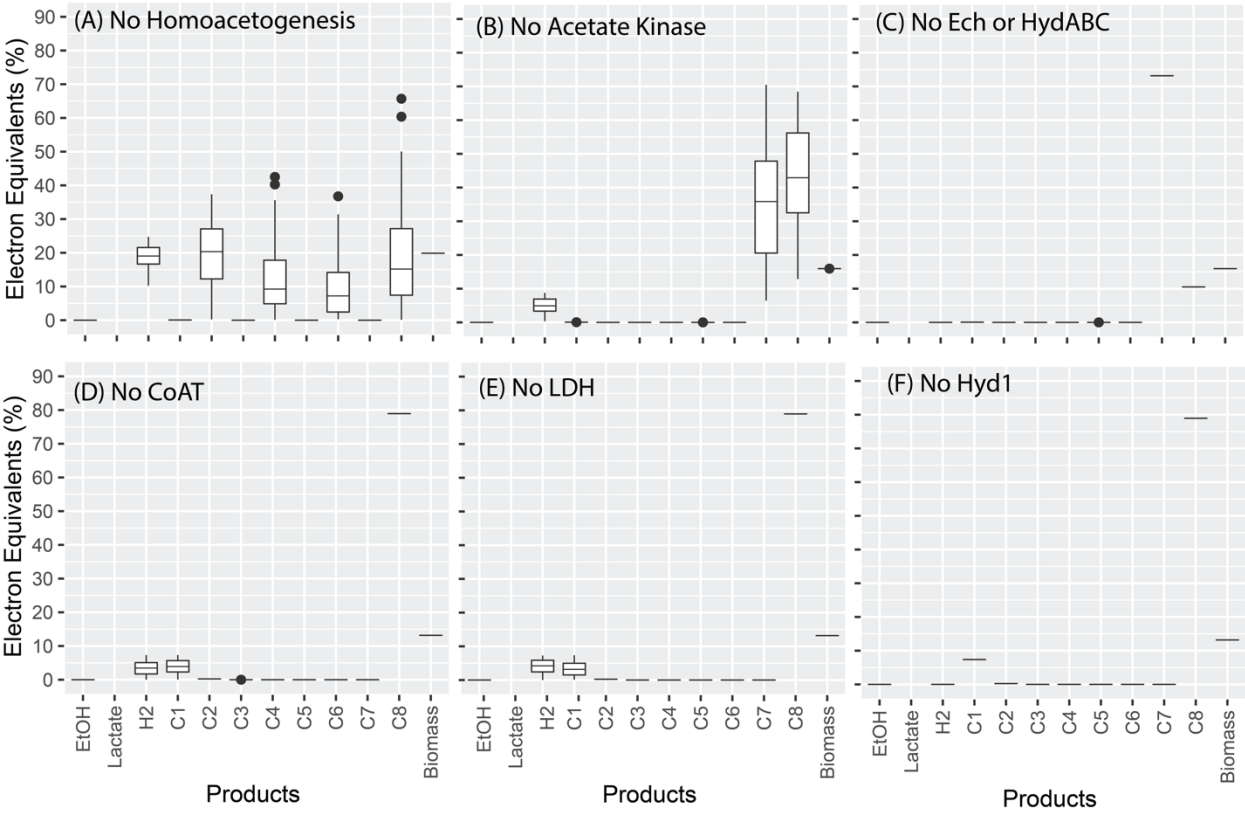

Supplement: FIG S2 [file mSystems.00755-20-sf002.pdf]
