# Supplementary material for: Diagnosing and Predicting Mixed-Culture Fermentations with Unicellular and Guild-Based Metabolic Models
Source: mSystems. 2020 Sep 29;5(5):e00755-20. doi: 10.1128/mSystems.00755-20 (PMC7527139; doi:10.1128/mSystems.00755-20)
Supplement: TEXT S1 [file mSystems.00755-20-s0001.docx]

**Supplementary Information for** ***Diagnosing and predicting mixed culture fermentations with unicellular and guild-based metabolic models***

*Matthew J. Scarborough,^1,2*^ Joshua J. Hamilton,^3^ Elizabeth A. Erb,^4^ Timothy J. Donohue,^1,5^*

*and Daniel R. Noguera^1,2^*

1. The Great Lakes Bioenergy Research Center, UW-Madison, Madison, WI
2. Department of Civil and Environmental Engineering, UW-Madison, Madison, WI
3. Department of Biochemistry, UW-Madison, Madison, WI
4. Department of Chemical and Biological Engineering, UW-Madison, Madison, WI
5. Department of Bacteriology, UW-Madison, Madison, WI

*corresponding author e-mail: mscarbor@uvm.edu

**Examples of organisms for each functional guild**

SEOs include organisms that utilize sugars and elongate intermediate products through reverse β-oxidation, such as *Megasphaera* and *Caproicproducens.*^1, 2^ SFOs include genera such as *Lactobacillus* and *Bifidobacterium*, which can ferment hexoses and pentoses to lactate, acetate and ethanol.^3, 4^ HSFs include organisms that generate H_2_ while producing fermentation products, such as members of the *Coriobacteriaceae* family.^5^ LEOs include organisms that perform reverse β-oxidation with lactate, such as *Pseudoramibacter^6^* and *Ruminococcaceae* bacterium *CPB6.^7^* EEOs, such as *Clostridium kluyveri,*^8^ perform reverse β-oxidation with ethanol. HAOs produce acetate from H_2_ and CO_2_ and include species within the *Clostridium*, *Acetobacterium*, *Eubacterium,* and *Blautia.*^9-11^

**Formation of a simplified biomass equation**

Simulating biomass growth requires a stochiometric equation accounting for required biomass precursors. Bacteria differ in their cellular composition, but gram positive bacteria are often abundant in fermentation bioreactors ^7, 12-15^. We therefore assumed a biomass composition similar to the well-studied gram positive bacterium *B. subtilis*, which contains proteins, nucleic acids, lipids, peptidoglycan, and lipoteichoic acid ^16, 17^. Stoichiometric coefficients for each precursor were calculated based on established pathways for synthesis of biomass components ^18^. We converted the stoichiometry for biomass production into 17 precursors contained in the iFerment215 and iFermGuilds789: α-ketoglutarate (aKG), oxaloacetate (OAA), glucose-6-phosphate (G-6-P), glucose-3-phosphate (G-3-P), 3-phosphoglycerate (3-PG), pyruvate, phosphoenolpyruvate, acetyl-CoA, erythrose-4-phosphate, ribulose-5-phosphate, ATP, NADPH, NAD+, ammonium, sulfate, and H_2_O.

Based on established amino acid biosynthesis pathways, we calculated the stoichiometry for synthesis of each amino acid. The final stoichiometry for each amino acid synthesis pathway is provided in **Supplementary Data File 3**. The synthesis of proteins from amino acids requires the equivalent of 4 mol ATP per mol amino acid incorporated into a protein. Aminoacyl-tRNA synthetase uses the equivalent of 2 mol ATP to load an amino acid into a tRNA,^19^ and 2 GTP are hydrolyzed by elongation factors (creating 2 GDP) for each peptide bond formed.^20^ Therefore, in addition to the ATP required to synthesize the amino acid, it was assumed that 4 mol ATP are required for each mol of amino acid incorporated into biomass.

RNA and DNA are formed by the polymerization of four different nucleoside triphosphates (NTPs: ATP, GTP, UTP, CTP) and four different deoxy-nucleoside triphosphates (dNTPs: dATP, dGTP, dTTP, dCTP), respectively. We assumed phosphotidylethanolamine as a representative lipid, which is the most abundant lipid reported in *B. subtilis* ^21^. Palmitate (C16:0), which is representative of the average size of fatty acid contained in *B. subtilis* lipids ^21^, was assumed to be the sole fatty acid incorporated into phosphotidylethanolamine. We assumed peptidoglycan is constructed of subunits containing N-acetylglucosamine, N-acetylmuramic acid, and a pentapeptide consisting of 1 L-alanine, D-glutamine, L-Lysine, and two D-alanine. We also assumed that the peptidoglycan subunits contain di-trans,octa-cis-undecaprenyl phosphate. Key metabolic intermediates of peptidoglycan synthesis include UDP-N-acetyl-α−glucosamine, isopentenyl diphosphate, and di-trans,octa-cis-undecaprenyl phosphate. Lipoteichoic acid (LTA) is a major component of gram positive cell walls consisting of a glycerol backbone and varying sugar units that is attached to the cell membrane via glycolipids. Type 1 LTA is widespread and found in many Firmicutes ^22, 23^. Production of LTA relies on several precursors, including G-6-P, an L-1-phosphaidyl-sn-glycerol (an intermediate of phospholipid synthesis), UDP-N-acetyl-α−glucosamine, and D-alanine.

We then determined the mmol of each precursor needed to produce one gram of dry cell weight (gDCW) by multiplying the stoichiometryby the mmol gDCW^-1^ (**Supplementary Data File 3**). This yielded the stoichiometry shown in the **Supplementary Data File 3 – Stoichiometric Matrix**. Minor modifications were made to the precision of calculated stoichiometric coefficients (**Supplementary Data File 1 – Biomass Equation**) to allow the model to achieve mass balance with biomass coproducts. The resulting biomass equation, based on 1 mmol of biomass having a mass of 1 gDCW, is shown below:

1.17 aKG + 2.06 OAA + 0.26 G-6-P + 1.58 G-3-P + 1.31 3-PG + 4.33 Pyruvate + 0.92 PEP + 3.06 Acetyl-CoA + 0.40 E-4-P + 0.35 R-5-P + 36.0 ATP + 19.39 NADPH + 1.10 NAD^+^ + 8.62 Ammonium + 0.21 Sulfate + 7.57 H_2_O ↔ 1 Biomass + 0.37 Fumarate + 0.43 Acetate + 0.29 Formate + 1.6 NAD^+^ + 19.4 NADP^+^ + 34.6 ADP + 1.4 AMP + 31.88 Pi + 4.74 PPi + 1.10 NADH + 3.54 CO_2_ + 10.13 H^+^ + 3.06 CoA

**Transport Constraints used in metabolic models**

To describe bioenergetic requirements for transport, we considered contributions of the electrochemical, pH, and concentration gradients across the cell envelope (**Eq. S1**).^24^ This predicts the energy required to transport a product from the interior of the cell to the extracellular space as represented by Equation 1, where R is the ideal gas constant (8.315 x 10^-3^ kJ mol^-1^ K^-1^), T is the temperature (K), [X]_ext_ is the concentration of product in the bulk liquid (M), [X]_int_ is the concentration of product inside a cell (M), $\Delta\psi$ is the difference in electric potential across the membrane (mV), c_j_ is the net charge transported from inside to outside the cell, F is Faraday’s constant (0.096485 kJ mV^-1^ mol^-1^), h_j_ is the number of protons transported across the membrane, and $\Delta pH$ (standard pH units) is the difference in pH between the intracellular and extracellular environments. We assumed an intracellular pH of 7.0 and an extracellular pH of 5.5 for all modeling scenarios, consistent with experimentational observations of mixed culture fermentation reactors^25^ and previously published models.^26^ Based on these conditions, we modeled the transport of unprotonated forms of products based on the pK_A_ values of carboxylic acid end products. Based on previous metabolic modeling approaches,^27^ we estimated the $\Delta\psi$ as shown in **Equation S2**.

${\Delta G}_{t}=RTln\left( \frac{{[X]}_{ext}}{\left[ X \right]_{int}} \right)+\Delta\psi c_{j}F-2.3h_{j}RT\Delta pH$ **(Eq. S1**)

$\Delta\psi=33.3\Delta pH-143.33$ **(Eq. S2**)

As such, the $\Delta\psi$ was equal to -93.4mV which is similar to potentials measured in other fermenting cultures.^28^ We further constrained intracellular product concentrations to 10 mM, consistent with previous studies^26^ and extracellular product concentrations to experimentally measured values obtained from the bulk liquid of a MCFA-producing mixed culture fermentation reactor.^29^ These bioenergetic calculations were then used to impose additional constraints on individual models, requiring that ATP hydrolysis for transport of end products be deducted from the gross ATP produced by the cell. This results in a net ATP yield that is a proxy for cell growth.

**Impacts of reaction knockouts on predicted products from lactate**

To assess the impacts of individual reaction on the predicted products of lactate consumption, we additively knocked out reactions in the iFermCell215 model. After knocking out homoaectogenesis, iFermCell215 predicted a mixture of C2, C4, C6, C8 and H_2_ as final products (**Fig. S2A**). Without acetate kinase, production of heptanoate (C7) and C8 is predicted (**Fig. S2B**). Without energy conserving hydrogenases (Ech and HydABC), the model predicted C7 as the most abundant product (**Fig S2B**). Knocking out CoAT results in predicted production of C8, formate, and H_2_ (**Fig. S2D**) and similar predictions were made after knocking out an NADH-dependent lactate dehydrogenase which required the model to use an electron-confurcating lactate dehydrogenase (**Fig S2E**). Finally, by knocking out Hyd1 (and all H2 production), the model predicted C8 and C1 as the products (**Fig S2F**). In total, these results suggest that excluding homoacetogensis, acetate kinase, and energy-conserving hydrogenases may improve production of C8.

**References**

1. Marounek, M.; Fliegrova, K.; Bartos, S., Metabolism and Some Characteristics of Ruminal Strains of Megasphaera-Elsdenii. *Appl. Environ. Microbiol.* **1989,** *55*, (6), 1570-1573.

2. Kim, B. C.; Seung Jeon, B.; Kim, S.; Kim, H.; Um, Y.; Sang, B. I., Caproiciproducens galactitolivorans gen. nov., sp. nov., a bacterium capable of producing caproic acid from galactitol, isolated from a wastewater treatment plant. *Int. J. Syst. Evol. Microbiol.* **2015,** *65*, (12), 4902-8.

3. Ganzle, M. G.; Follador, R., Metabolism of oligosaccharides and starch in lactobacilli: a review. *Front. Microbiol.* **2012,** *3*, 340.

4. Kandler, O., Carbohydrate metabolism in lactic acid bacteria. *Antonie Van Leeuwenhoek* **1983,** *49*, (3), 209-24.

5. Dewhirst, F. E.; Paster, B. J.; Tzellas, N.; Coleman, B.; Downes, J.; Spratt, D. A.; Wade, W. G., Characterization of novel human oral isolates and cloned 16S rDNA sequences that fall in the family Coriobacteriaceae: description of olsenella gen. nov., reclassification of Lactobacillus uli as Olsenella uli comb. nov. and description of Olsenella profusa sp. nov. *Int. J. Syst. Evol. Microbiol.* **2001,** *51*, (Pt 5), 1797-804.

6. Holdeman, L. V.; Elizabeth, C. P.; Moore, W. E. C., Amended description of Ramibacterium alactolyticum Prevot and Taffanel with propoosal of a neotype strain. *Int. J. Syst. Bacteriol.* **1967,** *17*, (4), 323-341.

7. Zhu, X.; Zhou, Y.; Wang, Y.; Wu, T.; Li, X.; Li, D.; Tao, Y., Production of high-concentration n-caproic acid from lactate through fermentation using a newly isolated Ruminococcaceae bacterium CPB6. *Biotechnol Biofuels* **2017,** *10*, 102.

8. H. A. Barker, M. D. K., and B.T. Borenson, The synthesis of butyric and caproic acid from ethanol and acetate by Clostridium

Kluyveri. *Proc. Natl. Acad. Sci. U. S. A.* **1945,** *31*, (12), 374-381.

9. Henderson, G.; Naylor, G. E.; Leahy, S. C.; Janssen, P. H., Presence of Novel, Potentially Homoacetogenic Bacteria in the Rumen as Determined by Analysis of Formyltetrahydrofolate Synthetase Sequences from Ruminants. *Appl. Environ. Microbiol.* **2010,** *76*, (7), 2058-2066.

10. Hugenholtz, J.; Ljungdahl, L. G., Metabolism and energy generation in homoacetogenic clostridia. *FEMS Microbiol. Rev.* **1990,** *7*, (3-4), 383-9.

11. Balch, W. E.; Schoberth, S.; Tanner, R. S.; Wolfe, R. S., Acetobacterium, a New Genus of Hydrogen-Oxidizing, Carbon Dioxide-Reducing, Anaerobic Bacteria. *Int. J. Syst. Bacteriol.* **1977,** *27*, (4), 355-361.

12. Andersen, S. J.; De Groof, V.; Khor, W. C.; Roume, H.; Props, R.; Coma, M.; Rabaey, K., A Clostridium Group IV Species Dominates and Suppresses a Mixed Culture Fermentation by Tolerance to Medium Chain Fatty Acids Products. *Front Bioeng Biotechnol* **2017,** *5*, 8.

13. Kucek, L. A.; Nguyen, M.; Angenent, L. T., Conversion of L-lactate into n-caproate by a continuously fed reactor microbiome. *Water Res.* **2016,** *93*, 163-171.

14. Zhu, X.; Tao, Y.; Liang, C.; Li, X.; Wei, N.; Zhang, W.; Zhou, Y.; Yang, Y.; Bo, T., The synthesis of n-caproate from lactate: a new efficient process for medium-chain carboxylates production. *Sci. Rep.* **2015,** *5*, 14360.

15. Weimer, P. J.; Nerdahl, M.; Brandl, D. J., Production of medium-chain volatile fatty acids by mixed ruminal microorganisms is enhanced by ethanol in co-culture with Clostridium kluyveri. *Bioresour. Technol.* **2015,** *175*, 97-101.

16. Henry, C. S.; Zinner, J. F.; Cohoon, M. P.; Stevens, R. L., iBsu1103: a new genome-scale metabolic model of Bacillus subtilis based on SEED annotations. *Genome Biol.* **2009,** *10*, (6), R69.

17. Oh, Y. K.; Palsson, B. O.; Park, S. M.; Schilling, C. H.; Mahadevan, R., Genome-scale reconstruction of metabolic network in Bacillus subtilis based on high-throughput phenotyping and gene essentiality data. *J. Biol. Chem.* **2007,** *282*, (39), 28791-9.

18. Caspi, R.; Billington, R.; Ferrer, L.; Foerster, H.; Fulcher, C. A.; Keseler, I. M.; Kothari, A.; Krummenacker, M.; Latendresse, M.; Mueller, L. A.; Ong, Q.; Paley, S.; Subhraveti, P.; Weaver, D. S.; Karp, P. D., The MetaCyc database of metabolic pathways and enzymes and the BioCyc collection of pathway/genome databases. *Nucleic Acids Res.* **2016,** *44*, (D1), D471-80.

19. Woese, C. R.; Olsen, G. J.; Ibba, M.; Soll, D., Aminoacyl-tRNA synthetases, the genetic code, and the evolutionary process. *Microbiol. Mol. Biol. Rev.* **2000,** *64*, (1), 202-36.

20. Alberts, B.; Johnson, A.; Lewis, J.; Raff, M.; Roberts, K.; Walter, P., From RNA to Protein. In *Mol. Biol. Cell*, 4th ed.; Garland Science: New York, 2002.

21. de Mendoza, D.; Schujman, G. E.; P, A., Biosynthesis and function of membrane lipids. In *Bacillus subtilis and its closest relatives*, Sonenshein, A. L.; Hoch, J. A.; Losick, R., Eds. American Society for Microbiology: Washington, D.C., 2002; pp 21-41.

22. Brown, S.; Santa Maria, J. P., Jr.; Walker, S., Wall teichoic acids of gram-positive bacteria. *Annu. Rev. Microbiol.* **2013,** *67*, 313-36.

23. Heptinstall, S.; Archibald, A. R.; Baddiley, J., Teichoic acids and membrane function in bacteria. *Nature* **1970,** *225*, (5232), 519-21.

24. Hamilton, J. J.; Dwivedi, V.; Reed, J. L., Quantitative assessment of thermodynamic constraints on the solution space of genome-scale metabolic models. *Biophys. J.* **2013,** *105*, (2), 512-22.

25. Scarborough, M. J.; Lynch, G.; Dickson, M.; McGee, M.; Donohue, T. J.; Noguera, D. R., Increasing the economic value of lignocellulosic stillage through medium-chain fatty acid production. *Biotechnology for Biofuels* **2018,** *11*, (1), 200.

26. Rodriguez, J.; Kleerebezem, R.; Lema, J. M.; van Loosdrecht, M. C. M., Modeling product formation in anaerobic mixed culture fermentations. *Biotechnol. Bioeng.* **2006,** *93*, (3), 592-606.

27. Henry, C. S.; Broadbelt, L. J.; Hatzimanikatis, V., Thermodynamics-based metabolic flux analysis. *Biophys. J.* **2007,** *92*, (5), 1792-805.

28. Kashket, E. R., The proton motive force in bacteria: a critical assessment of methods. *Annu. Rev. Microbiol.* **1985,** *39*, 219-42.

29. Scarborough, M. J.; Lawson, C. E.; Hamilton, J. J.; Donohue, T. J.; Noguera, D. R., Metatranscriptomic and Thermodynamic Insights into Medium-Chain Fatty Acid Production Using an Anaerobic Microbiome. *mSystems* **2018,** *3*, (6).
