# Supplementary material for: Diagnosing and Predicting Mixed-Culture Fermentations with Unicellular and Guild-Based Metabolic Models
Source: mSystems. 2020 Sep 29;5(5):e00755-20. doi: 10.1128/mSystems.00755-20 (PMC7527139; doi:10.1128/mSystems.00755-20)
Supplement: TABLE S1 [file mSystems.00755-20-st001.docx]

| **Name** | **Abbreviation** | **Formula** | **eeq mol^-1^** | **ΔG_f_^0^ (kJ mol^-1^)** |
| --- | --- | --- | --- | --- |
| Glucose | glc | C_6_H_12_O_6_ | 24 | -913 |
| Xylose | xyl | C_5_H_10_O_5_ | 20 | -753 |
| Glucan (stachyose) | glc4 | C_24_H_48_O_24_ | 96 | -2,906 |
| Xylan (tetra-arabinofuranoside) | xyl4 | C_20_H_34_O_17_ | 90 | -2,265 |
| Glycerol | glyc | C_3_H_8_O_3_ | 14 | -486 |
| Ethanol | etoh | C_2_H_6_O | 12 | -182 |
| Lactate | lac | C_3_H_6_O_3_^-^ | 12 | -515 |
| Formate | for | C_1_H_1_O_2_^-^ | 2 | -351 |
| Acetate | ac | C_2_H_3_O_2_^-^ | 8 | -369 |
| Propionate | ppa | C_3_H_5_O_2_^-^ | 14 | -356 |
| Butyrate | but | C_4_H_7_O_2_^-^ | 20 | -353 |
| Valerate | pta | C_5_H_9_O_2_^-^ | 26 | -343 |
| Hexanoate | hxa | C_6_H_11_O_2_^-^ | 32 | -336 |
| Heptanoate | hpta | C7H_13_O_2_^-^ | 38 | -329 |
| Octanoate | octa | C8H_15_O_2_^-^ | 44 | -322 |
| Hydrogen | h2 | H_2_ | 2 | 0 |
| Hydrogen ions | h | H^+^ | 0 | 0 |
| Carbon dioxide | co2 | CO_2_ | 0 | -386 |
| Water | h2o | H_2_O | 0 | -237 |
