# Supplementary material for: Diagnosing and Predicting Mixed-Culture Fermentations with Unicellular and Guild-Based Metabolic Models
Source: mSystems. 2020 Sep 29;5(5):e00755-20. doi: 10.1128/mSystems.00755-20 (PMC7527139; doi:10.1128/mSystems.00755-20)
Supplement: TABLE S2 [file mSystems.00755-20-st002.docx]

| **Compound** | **meeq/hr** | **% of eeq** |
| --- | --- | --- |
| **Substrates** | | |
| Glucose | 0.300 | 6.1% |
| Xylose | 2.635 | 53.9% |
| Glucans | 0.780 | 16.0% |
| Xylans | 0.731 | 15.0% |
| Lactate | 0.007 | 0.1% |
| Glycerol | 0.433 | 8.9% |
| **Products** | | |
| Ethanol | 0.112 | 2.3% |
| Formate | 0.000 | 0.0% |
| Acetate | 0.625 | 12.8% |
| Propionate | 0.000 | 0.0% |
| Butyrate | 1.755 | 35.9% |
| Valerate | 0.000 | 0.0% |
| Hexanoate | 1.615 | 33.0% |
| Heptanoate | 0.000 | 0.0% |
| Octanoate | 0.161 | 3.3% |
| H_2_ | 0.383 | 7.8% |
| Biomass | 0.236 | 4.8% |
